# SERBP1 Promotes Stress Granule Clearance by Regulating 26S Proteasome Activity and G3BP1 Ubiquitination and Protects Male Germ Cells from Thermostimuli Damage

**DOI:** 10.34133/research.0091

**Published:** 2023-04-12

**Authors:** Fengli Wang, Lingjuan Wang, Shiming Gan, Shenglei Feng, Sijin Ouyang, Xiaoli Wang, Shuiqiao Yuan

**Affiliations:** ^1^ Institute of Reproductive Health, Tongji Medical College, Huazhong University of Science and Technology, Wuhan 430030, China.; ^2^ Laboratory of Animal Center, Huazhong University of Science and Technology, Wuhan 430030, China.

## Abstract

Stress granules (SGs) are membraneless cytoplasmic condensates that dynamically assemble in response to various stressors and reversibly disassemble after stimulus removal; however, the mechanisms underlying SG dynamics and their physiological roles in germ cell development are elusive. Here, we show that SERBP1 (SERPINE1 mRNA binding protein 1) is a universal SG component and conserved regulator of SG clearance in somatic and male germ cells. SERBP1 interacts with the SG core component G3BP1 and 26S proteasome proteins PSMD10 and PSMA3 and recruits them to SGs. In the absence of SERBP1, reduced 20S proteasome activity, mislocalized valosin containing protein (VCP) and Fas associated factor family member 2 (FAF2), and diminished K63-linked polyubiquitination of G3BP1 during the SG recovery period were observed. Interestingly, the depletion of SERBP1 in testicular cells in vivo causes increased germ cell apoptosis upon scrotal heat stress. Accordingly, we propose that a SERBP1-mediated mechanism regulates 26S proteasome activity and G3BP1 ubiquitination to facilitate SG clearance in both somatic and germ cell lines.

## Introduction

In eukaryotic cells, cytoplasmic mRNA ribonucleoprotein granules represent a conserved and unique machinery that governs many aspects of RNA metabolism, thus controlling gene expression [1]. Stress granules (SGs), one prominent type of ribonucleoprotein granule, are dynamic, reversible, and membraneless biomolecular condensates assembled in response to various stressors, including heat shock and arsenite [2]. SG is a unique protective and adaptive mechanism that occurs to prevent pivotal mRNA degradation when cells encounter acute unfavorable conditions. The formation of SGs is triggered by translation suppression; therefore, the compositions of SGs include translationally arrested mRNAs, translation initiation factors, a subtype of RNA-binding proteins, and non-RNA binding proteins that are recruited through protein–protein interactions [3].

The dynamic and reversible organization implies that SGs assemble upon stimuli induction but disassemble after stressor removal, modulated by various posttranslational modifications as well as numerous adenosine triphosphate-dependent ribonucleoprotein or protein remodeling complexes [3,4]. An analysis of granule assembly steps reveals that core formation is an early event followed by the recruitment of shell components, while disassembly is also a stepwise process with shell dissipation followed by core collapse [5]. Unbalanced SG dynamics or impaired SG clearance that leads to persistent and toxic aggregates could contribute to human diseases, especially neurodegeneration, including amyotrophic lateral sclerosis and frontotemporal lobar degeneration [6,7]. The molecular mechanisms that govern SG assembly in response to various stressors and in different model systems have been extensively studied for years, whereas less attention has been given to the SG disassembly process. There is a marked link between SGs and ubiquitin-dependent proteasome system activity, whose inhibition induces the formation of SGs [8]. A recent study revealed that inhibition of the ubiquitin-activating enzyme and the 26S proteasome could impair the clearance of arsenite- and heat-induced SGs, directly affecting the ubiquitin-dependent proteasome system and ubiquitin pathway in SG dynamics and turnover [9]. Accordingly, factors such as ZFAND1 (zinc finger AN1-type containing 1), which mediates 26S proteasome recruitment, could contribute to SG clearance [10]. Moreover, VCP (valosin containing protein), an AAA-adenosine triphosphatase ubiquitin segregase that is involved in both degradative and nondegradative ubiquitin signaling, indispensably facilitates the clearance of SGs [11,12]. Excitingly, 2 recent landmark works systematically analyzed the SG and non-SG ubiquitinome in response to different stressors through an unbiased proteomic approach and highlighted the significance of heat shock-specifically induced ubiquitination of the SG core factor G3BP1. Targeting ubiquitinated G3BP1 by VCP in conjunction with the adaptor protein FAF2 (Fas associated factor family member 2) ultimately results in SG disassembly, revealing the mechanism governing SG clearance [13,14]. Although these works provide a comprehensive understanding of ubiquitinated proteins during both the assembly and disassembly stages, a series of unknown questions remain, such as how the ubiquitin system mediates stress-specific responses and what other factors modulate the ubiquitin system.

Mammalian spermatogenesis consists of highly complex and thermosensitive biological processes in the testis, which raises the possibility that heat-induced SG dynamics and turnover function in male germ cell survival against thermostimuli stress [15]. Heat shock stress can induce SGs in spermatogenic cells, such as spermatogonia and spermatocytes [16]. Of note, in addition to containing the common SG-related proteins identified in somatic cells, SGs in male germ cells comprise many specific factors, such as DAZL (deleted in azoospermia like) and BOULE (boule homolog, RNA binding protein) [16,17]. More importantly, the testis-specific protein MAGE-B2 transcriptionally fine-tunes G3BP levels in vivo to modulate germ cell SGs, while overexpression of G3BP results in hypersensitivity to heat stress and reduced fertility, providing direct evidence that SG induction or tolerance contributes to spermatogonial cell survival against heat shock stress [18]. However, the physiological and cellular functions of SGs induced in male germ cells and the molecular mechanism of spermatogenesis under adverse temperature fluctuations remain elusive.

SERBP1 (SERPINE1 mRNA binding protein 1) is a conserved RNA binding protein comprising arginine-glycine (RG) and arginine-glycine-glycine (RGG) repeat motifs for mRNA targeting and is involved in both physiological cellular processes, such as cell division and DNA damage response, and certain cancers through mRNA translation regulation [19–21]. In addition, SERBP1 can recognize G-rich RNA sequences at the C-terminus and undergo liquid–liquid phase separation, mediated by the addition of salt and RNA, and RGG-rich binding motifs are necessary for the efficient formation of condensed phases [22]. Of note, SERBP1 colocalizes with the typical SG marker TIA-1 (TIA1 cytotoxic granule associated RNA binding protein) upon arsenite treatment [23]. However, the functional interpretation beyond recruitment to SGs and the molecular mechanisms underlying its particular role in SG dynamics have not been explored.

Here, we report that SERBP1 is required for the efficient clearance of arsenite- and heat shock-induced SGs in mammalian cells and protects male germ cells against heat shock-induced apoptosis in vivo. We showed that SERBP1 interacted with 26S proteasomes, such as PSMA3 and PSMD10, acting as an upstream modulator to manipulate 26S proteasomes to mediate SG clearance. We further demonstrated that SERBP1 mediates the recruitment of VCP and the 20S proteasome to SGs and that this is a prerequisite for efficient SG clearance during recovery from arsenite stress and heat shock. Specifically, in the context of heat shock, SERBP1 modulates the ubiquitination level of G3BP1, the central protein within the SG RNA–protein network, facilitating the collapse of interactions among SG factors. Interestingly, we also found that SERBP1 is localized within SGs in mouse male germ cells upon arsenite exposure in vivo, and the depletion of SERBP1 in the mouse testis causes increased sensitivity to heat stress characterized by the increased loss of male germ cells. Collectively, these data defined SERBP1 as a universal SG component and uncovered a novel mechanism of its regulatory role in SG disassembly and its critical function in protecting thermosensitive male germ cells from apoptosis under heat shock stress.

## Results

### SERBP1 serves as a universal SG component and is dependent on its RGG-rich domain

**Fig. 1.**
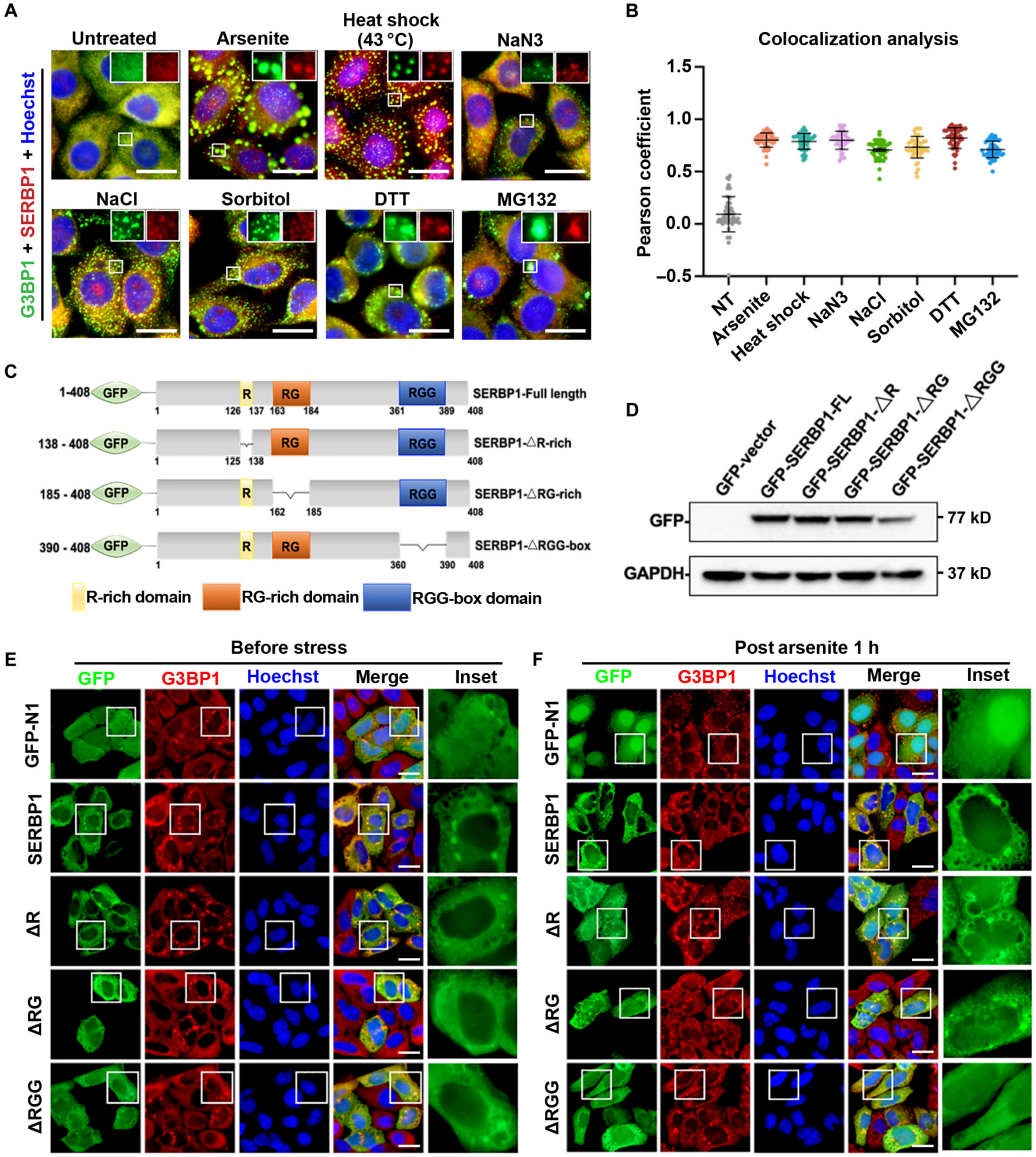
SERBP1 is a universal SG component and is dependent on its RGG-rich domain. (A) Confocal images of HeLa cells treated with different SG induction stressors and stained with anti-G3BP1 (green) and anti-SERBP1 (red) antibodies and counterstained with Hoechst (blue) are shown. Stressors include arsenite treatment, heat shock (43 °C), endoplasmic reticulum stress (DTT treatment), osmotic stress (200 mM NaCl treatment), osmotic and oxidative stress (sorbitol treatment), proteasome inhibition (MG132 treatment), and mitochondrial stress (NaN3). White boxes indicate the enlarged region of interest (ROI) and are displayed in separate channels (top right). Scale bars = 10 μm (main image). (B) Scatterplot representing the colocalization correlation between G3BP1 and SERBP1 signal in (A). Data are shown as means ± SD. Statistical significance was determined using an unpaired *t* test comparison. Each dot represents the Pearson coefficient analysis of a single ROI. Three experiments were performed independently. (C) Representative diagrams showing the domain organization of human SERBP1 fused with GFP, including full length, the R-rich deletion (ΔR), RG-rich deletion (ΔRG), and the RGG repeats deletion (ΔRGG). Numbers indicate amino acid order. R, arginine; RG, arginine-glycine; RGG, arginine-glycine-glycine. (D) Western blot analysis of lysates from HeLa cells transfected with GFP vector or GFP-tagged SERBP1 full length or different deletions (ΔR, ΔRG, and ΔRGG). The membrane was blotted with an anti-GFP antibody or glyceraldehyde-3-phosphate dehydrogenase antibody that serves as a loading control. (E and F) HeLa cells were transfected with GFP or GFP-tagged SERBP1 as indicated. At 36 h post-transfection, cells were treated with 0.5 mM sodium arsenite for 1 h and immunostained with anti-GFP and anti-G3BP1 and then counterstained with Hoechst. Cells at pre- (E) and 1-h post-arsenite treatment (F) are shown. Scale bars = 10 μm.

SERBP1, which belongs to the RNA binding protein family containing low-complexity RG- or RGG-repeat motifs, has been reported to be located in arsenite-induced SGs [23]. However, whether it is a universal SG component induced by other stimuli and what its potential involvement in SG metabolism might be remain unknown. To determine the recruitment of SERBP1 into SGs, we first subjected HeLa cells to various SG inducers and utilized specific antibodies to test the colocalization between SERBP1 and SG core protein G3BP1. The results showed that SERBP1 foci could be observed in the cytoplasm under all the detected stimuli, including arsenite treatment, heat shock (43 °C), endoplasmic reticulum stress (dithiothreitol [DTT] treatment), osmotic stress (200 mM NaCl treatment), osmotic and oxidative stress (sorbitol treatment), proteasome inhibition (MG132 treatment), and mitochondrial stress (NaN3). Notably, cytoplasmic SERBP1 foci showed a high degree of colocalization with G3BP1 in all stimulations (Fig. 1A and B and Fig. S1A and B), suggesting that SERBP1 is a universal SG component and is associated with SG formation under various stresses. Moreover, we observed that treatment with cycloheximide (a drug that stalls ribosomes by blocking translation elongation and inhibits SG assembly) could substantially disrupt the formation of SERBP1 and G3BP1 foci under different stimuli (Fig. S1C). These results indicate that SERBP1 could be generally recruited into SGs, and this recruitment does not display stress specificity.

Since RNA–protein interaction is the fundamental force promoting SG formation and modulating SG dynamics, we next asked whether the RNA binding domain of SERBP1 is required for mediating SERBP1 association with SGs. To this end, we first generated different SERBP1 mutants lacking R, RG, and RGG repeat motifs and transiently expressed them in HeLa cells (Fig. 1C and D). We then performed immunofluorescence assays to detect the colocalization of different truncated SERBP1 proteins with G3BP1 before and 1 h after arsenite treatment. The results showed that deletion of the RGG-rich motif substantially eliminated the formation of SERBP1-green fluorescent protein (GFP) foci upon SG induction, while R-rich or RG-rich deletion resulted in a obvious decrease in foci number colocalized with G3BP1 compared with that of wild-type (WT) SERBP1 (Fig. 1E and F), suggesting that the RGG-rich binding domain of SERBP1 is essential for the recruitment of SERBP1 into SGs under stimuli.

### SERBP1 interacts with G3BP1 but is independent of G3BP1 recruitment into SGs

**Fig. 2.**
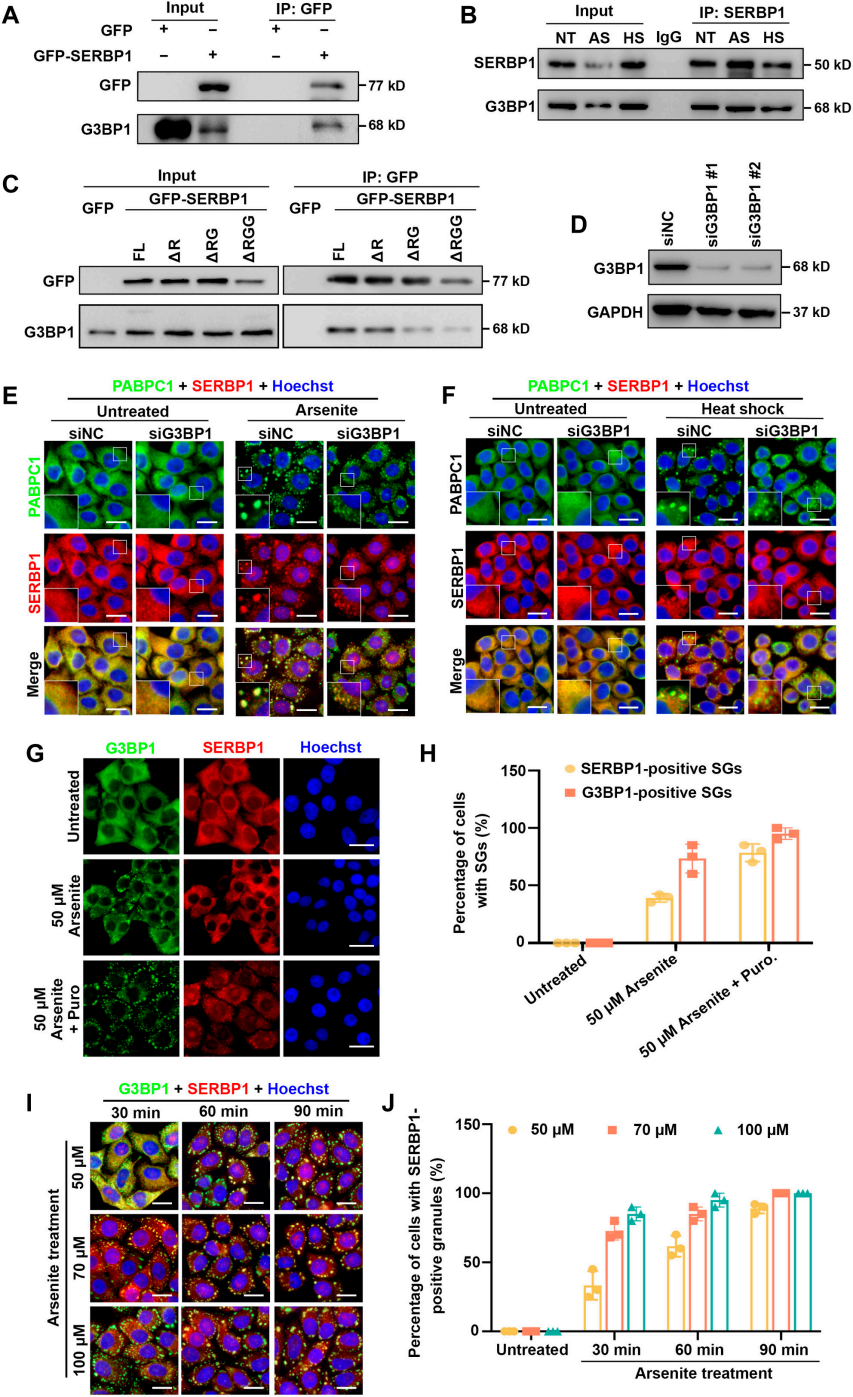
G3BP1 interacts with SERBP1 but not mediates its recruitment with SGs. (A) Representative images of a Western blot of lysates from the co-IP experiment are shown. HEK293 cells expressing C-terminally GFP-tagged SERBP1 or GFP empty vector were lysed and subjected to anti-GFP immunoprecipitation (IP). Input and IP samples were immunoblotted for GFP and G3BP1. (B) Representative images of a Western blot of lysates from the co-IP experiments are shown. HeLa cells were treated with 0.5 mM sodium arsenite for 1 h or cultured at 43 °C for 1 h, or not treated with any stress, and then subjected to anti-SERBP1 IP. The membrane was immunoblotted with SERBP1 and G3BP1 antibodies to detect protein levels in input and IP samples. NT, nontreated; AS, arsenite stress; HS, heat stress. (C) Images of a Western blot of lysates from co-IP experiments are shown. HEK293 cells expressing C-terminally GFP-tagged SERBP1 (full length or deletions) or GFP empty vector were lysed and then subjected to anti-GFP IP. Input and IP samples were immunoblotted for GFP and G3BP1. FL, full length; ΔR, R-rich deletion; ΔRG, RG-rich deletion; ΔRGG, RGG repeats deletion. (D) Representative images of immunoblot confirming G3BP1 depletion in HeLa cells transfected with a control siRNA (siNC) or with siRNAs targeting G3BP1 (siG3BP1 #1 and #2) are shown. (E and F) HeLa cells were transfected with the indicated siRNA targeting G3BP1 (siG3BP1) or nontargeted control (siNC) for 48 h and then subjected to sodium arsenite treatment (0.5 mM, 1 h) (E) or cultured at 43 °C for 1 h (F). The SERBP1 (red) and SG marker PABPC1 (green) were detected by immunofluorescence and visualized by a confocal microscopy. Scale bars = 10 μm. (G) Confocal images of HeLa cells stained with anti-G3BP1 (green) and anti-SERBP1 (red) antibodies and counterstained with Hoechst (blue) are shown. Cells were either untreated (upper panel), treated with 0.05 mM arsenite for 30 min (middle panel), or cotreated with both arsenite (0.05 mM) and puromycin (5 μg/ml) for 30 min (lower panel). Scale bars = 10 μm. (H) Quantification of the percentage of HeLa cells with either SERBP1-positive SGs or G3BP1-positive SGs in (G). Data are shown as means ± SD from 3 independent experiments. (I) Confocal images of HeLa cells stained with anti-G3BP (green) and anti-SERBP1 (red) antibodies and counterstained with Hoechst (blue) are shown. Cells were treated with increased concentrations of arsenite (50, 70, and 100 μM) for different incubation times (30, 60, and 90 min). Scale bars = 10 μm. (J) Quantification of the percentage of HeLa cells with SERBP1-positive in conditions tested in (H). Data are shown as means ± SD from 3 independent experiments.

Given that G3BP1 is a core component of SGs and is responsible for mediating protein–protein and protein–RNA interaction networks during SG assembly and dynamics, we next asked whether G3BP1 affects SERBP1 recruitment into SGs. To address this question, we first investigated the intrinsic interaction between the G3BP1 and SERBP1 proteins. Ectopically expressed GFP-tagged SERBP1 in 293T cells was immunoprecipitated, and endogenous G3BP1 was co-immunoprecipitated with GFP-SERBP1 (Fig. 2A). Consistently, we found that endogenous SERBP1 could interact with G3BP1 in the unperturbed condition and the stress conditions induced by arsenite stress and heat shock, respectively (Fig. 2B). Furthermore, we detected a slightly decreased G3BP1 binding ability to SERBP1 upon deletion of either the RG-rich or RGG-rich motif in SERBP1 (Fig. 2C), suggesting that the interaction between G3BP1 and SERBP1 is dependent on the RNA-binding motifs of SERBP1.

To further explore the association of G3BP1 and SERBP1 during SG assembly, we knocked down G3BP1 using specific small interfering RNAs (siRNAs) and visualized SERBP1 foci and SG formation in cells following arsenite exposure or heat shock treatment. A strongly reduced G3BP1 signal was observed in knockdown (KD) cells (Fig. 2D and Fig. S2A and B), whereas G3BP1 depletion did not block SG assembly, as evidenced by the accumulation of PABPC1 (poly(A) binding protein cytoplasmic 1) (another SG marker), but the SG foci became much smaller compared to those in control cells under arsenite or heat shock treatment (Fig. 2E and F). Interestingly, we observed SERBP1 foci colocalizing with PABPC1 even after G3BP1 depletion, indicating that the recruitment of SERBP1 into SGs is independent of the core protein G3BP1 (Fig. 2E and F).

Although G3BP1 depletion does not affect SERBP1 recruitment, the protein interaction between them encouraged us to examine the dynamic timing of SERBP1 and G3BP1 localization after SG induction. To dissect the steps of G3BP1 and SERBP1 recruitment into SGs, we treated cells with a suboptimal concentration of arsenite (50 μM) and detected their localization. After 1 h of arsenite treatment, ~70% of cells contained G3BP1 foci, whereas only a fraction of cells (~45%) had SERBP1 foci (Fig. 2G and H), indicating that the formation of SERBP1 granules occurred later than that of G3BP1 granules. In addition, cotreatment with arsenite and puromycin (a chemical that can induce polysome release) enhanced both G3BP1-positive and SERBP1-positive SG formation (Fig. 2G and H), suggesting that SERBP1 was wrapped in SGs after polysome disassembly. To further narrow down the process of SERBP1 granule formation, we approximately monitored G3BP1 and SERBP1 foci in cells treated with various concentrations of (50, 70, and 100 μM) arsenite at different time points. We observed a gradually increased percentage of cells with SERBP1-positive granules in a dose- and time-dependent manner and did not identify granules labeled only by SERBP1, which indicated that all SERBP1-positive granules contained G3BP1 (Fig. 2I and J). Additional treatment with puromycin in all these conditions could promote SERBP1 granule formation compared with the corresponding arsenite treatment (Fig. S2C and D). Together, these results suggest that SERBP1 is independently recruited to SGs with G3BP1 after polysome disassembly.

### SERBP1 is required for efficient clearance of arsenite- and heat shock-induced SGs

**Fig. 3.**
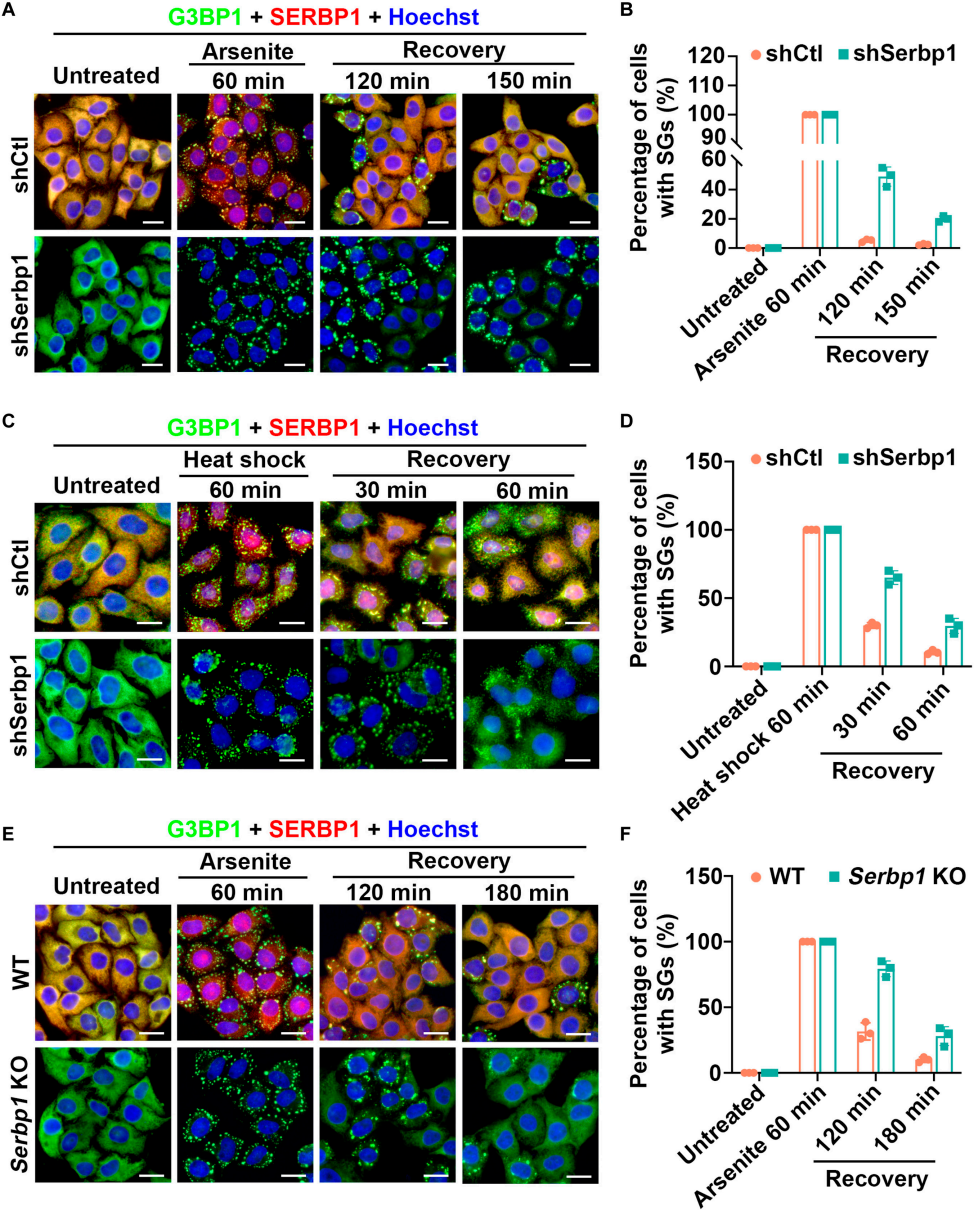
Depletion of SERBP1 delays the SG disassembly in both arsenite- and heat shock-induced SGs. (A) HeLa cells stably depleted SERBP1 (shSERBP1) using shRNA lentivirus or HeLa cells with control shRNA (shCtl) were treated with sodium arsenite (0.5 mM, 60 min) and then allowed to recover for 120 or 150 min. SERBP1 (red) and SG marker G3BP1 (green) were detected by immunofluorescence and visualized by confocal microscopy. Scale bars = 10 μm. (B) Quantitation of the percentage of cells with SGs in (A). Data are shown as means ± SD collected from 3 independent experiments with more than 150 cells per condition. (C) HeLa cells stably depleted SERBP1 (shSERBP1) using shRNA lentivirus or HeLa cells with control shRNA (shCtl) were cultured at 43 °C for 60 min and then allowed to recover for 30 or 60 min. SERBP1 (red) and SG marker G3BP1 (green) were detected by immunofluorescence and visualized by confocal microscopy. Scale bars = 10 μm. (D) Quantitation of the percentage of cells with SGs in (C). Data are shown as means ± SD collected from 3 independent experiments with more than 100 cells per condition. (E) WT HeLa cells or SERBP1 KO HeLa cells generated by using CRISPR/Cas9 strategy were treated with sodium arsenite (0.5 mM, 60 min) and then allowed to recover for 120 or 180 min. SERBP1 (red) and SG marker G3BP1 (green) were detected by immunofluorescence and visualized by confocal microscopy. Scale bars = 10 μm. (F) Quantitation of the percentage of cells with SGs in (E). Data are shown as means ± SD collected from 3 independent experiments with 150 cells per condition.

After characterizing SERBP1 recruitment into SGs induced by diverse stimuli, we next set out to elucidate the cellular function of SERBP1 in SG metabolism. To this end, we first employed specific short hairpin RNA (shRNA) to stably deplete the SERBP1 gene in HeLa cells (Fig. S3A to C). After treatment with 0.5 mM arsenite for 30 min, nearly all SERBP1 KD cells contained G3BP1-positive SGs, which was similar to the control cells (Fig. 3A and B). Intriguingly, in SERBP1 KD cells, we observed a large portion of persistent SGs at both 120 and 150 min after arsenite removal (recover phase), whereas most control cells had efficiently cleared the SGs by these time points (Fig. 3B). Consistently, the depletion of SERBP1 impaired the clearance of SGs induced by heat shock, as evidenced by over 20% of SERBP1 KD cells containing SGs compared with less than 10% of control cells after 60 min of recovery from heat shock stress (Fig. 3C and D). Moreover, we generated SERBP1 knockout (KO) cells using the CRISPR/Cas9 strategy to further confirm the disassembly defects of arsenite-induced SGs. Cells lacking SERBP1 possessed significantly delayed SG clearance (Fig. 3E and F), which was consistent with the results observed in SERBP1 KD cells. Taken together, these results indicate that SERBP1 is essential for the clearance of arsenite- and heat shock-induced SGs.

### SERBP1 binds to the 26S proteasome and manipulates proteasome activity

**Fig. 4.**
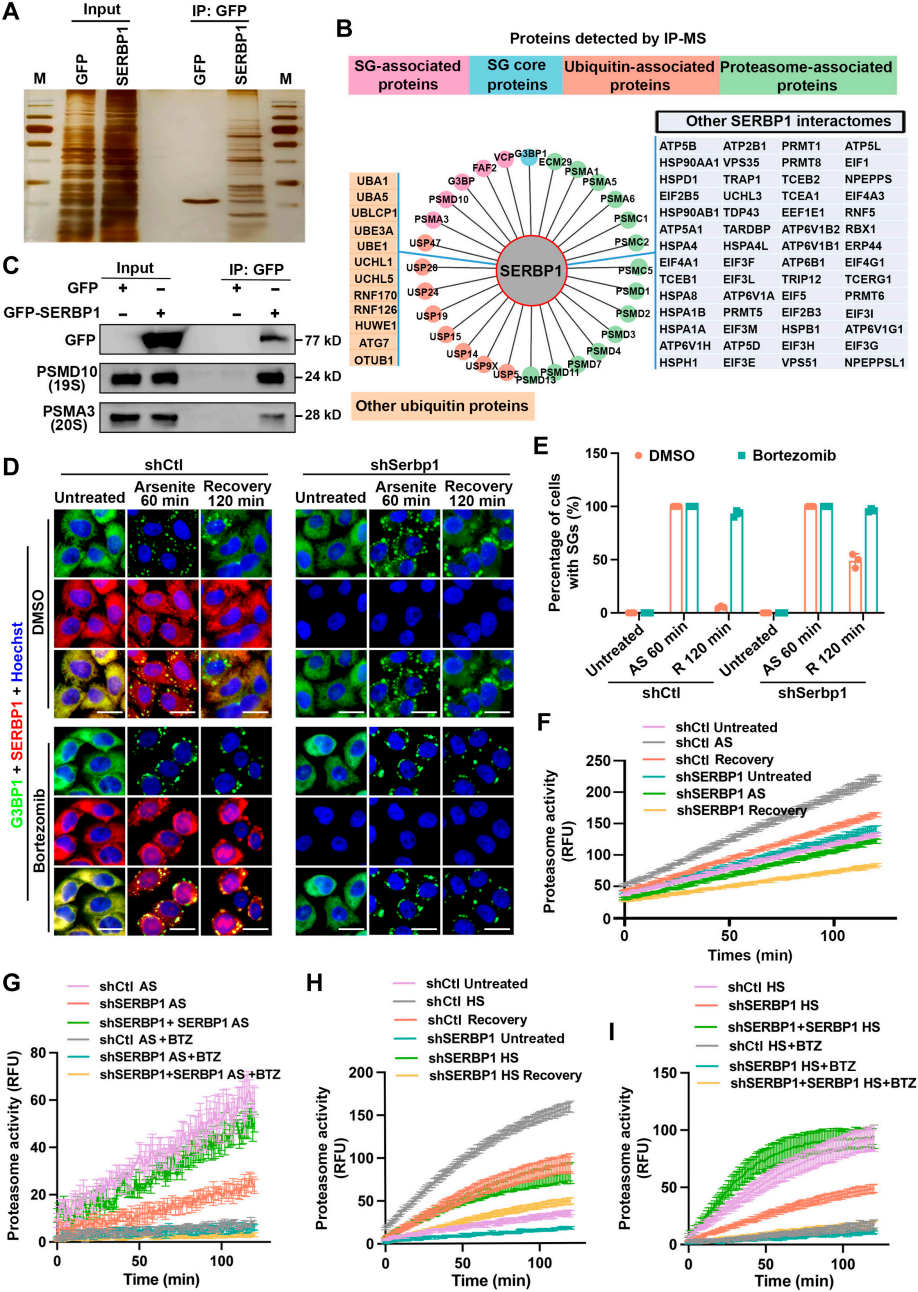
SERBP1 interacts with 26S proteasome subunits and manipulates its hydrolysis activity. (A) Silver staining image of SDS-PAGE gel-separated samples from the co-IP experiment is shown. HEK293 cells expressing C-terminally GFP-tagged SERBP1 or GFP empty vector were lysed and subjected to anti-GFP IP. Input and IP proteins were separated by SDS-PAGE gel and visualized by silver staining. (B) Integrated analysis of SERBP1 interactome from IP-MS data showed distinct clusters of SERBP1 interactors. (C) HEK293 cells expressing C-terminally GFP-tagged SERBP1 or GFP empty vector were lysed and then subjected to anti-GFP IP. Input and IP samples were immunoblotted for GFP, 20S subunit (PSMD10), and 19S subunit (PSMA3). (D) Confocal images of HeLa cells stained with anti-SERBP1 (red) and anti-G3BP1 (green) antibodies and counterstained with Hoechst (blue). HeLa cells stably depleted SERBP1 (shSerbp1) using shRNA lentivirus or HeLa cells with control shRNA (shCtl) were either cotreated with 0.5 mM sodium arsenite and dimethyl sulfoxide (DMSO) or cotreated with 0.5 mM sodium arsenite and 2 μg/ml BTZ for 60 min were immediately collected or allowed to recover for 120 min after drugs removal. Scale bars = 10 μm. (E) Quantitation of the percentage of cells with SGs in (D). Data are shown as means ± SD collected from 3 independent experiments with more than 150 cells per condition. AS, arsenite-treated; R, recovery. (F) 26S proteasomal activity measurement using Suc-LLVY-AMC substrate. HeLa cells stably depleted SERBP1 (shSerbp1) using shRNA lentivirus or HeLa cells with control shRNA (shCtl) were treated with 0.5 mM arsenite for 60 min (AS), allowed to recover for 120 min after drug removal (Recovery), and then lysed, and AMC signal was detected with excitation wavelength 360 nm and emission wavelength 460 nm on a microplate reader to record for 120 min. (G) HeLa cells stably depleted SERBP1 (shSerbp1) using shRNA lentivirus or SERBP1 depleted HeLa cells transfected with plasmid encoding SERBP1, or HeLa cells with control shRNA (shCtl) were treated with 0.5 mM arsenite for 60 min (AS), and allowed to recover for 120 min after drug removal (Recovery), while each group was cotreated with BTZ as corresponding negative control. Cells were then lysed and AMC signal was detected with excitation wavelength 360 nm and emission wavelength 460 nm on a microplate reader to record for 120 min. (H) HeLa cells stably depleted SERBP1 (shSerbp1) using shRNA lentivirus or HeLa cells with control shRNA (shCtl) were cultured at 43 °C for 60 min (HS), allowed to recover for 30 min (HS recovery), and then lysed, and AMC signal was detected with excitation wavelength 360 nm and emission wavelength 460 nm on a microplate reader to record for 120 min. (I) HeLa cells stably depleted SERBP1 (shSERBP1) using shRNA lentivirus or SERBP1 depleted HeLa cells transfected with plasmid encoding SERBP1, or HeLa cells with control shRNA (shCtl) were cultured at 43 °C for 60 min (HS), and allowed to recover for 30 min, while each group was cotreated with BTZ as corresponding negative control. Cells were then lysed and AMC signal was detected with excitation wavelength 360 nm and emission wavelength 460 nm on a microplate reader to record for 120 min. RFU, relative fluorescence units.

To obtain insight into the molecular mechanisms underlying delayed SG clearance in SERBP1-deficient cells, we mapped out a comprehensive picture of SERBP1 binding patterns using an unbiased proteomics approach. We identified the interactome of GFP-SERBP1 through affinity purification followed by mass spectrometry (Table S1). Silver staining displayed specific bands of interactors immunoprecipitated by GFP-SERBP1 compared with an empty vector (Fig. 4A). We performed functional enrichment analysis of SERBP1-interacting proteins using the STRING (search tool for the retrival of interacting genes/proteins) database and unexpectedly observed overrepresentation of many 26S proteasome subunit components and ubiquitination process-related factors (Fig. 4B). In addition, the interactome highlighted interactions between SG-associated proteins, including the core protein G3BP1, and the disassembly factors VCP and FAF2 (Fig. 4B). Interactions between SERBP1 and 26S proteasome proteins PSMD10 (20S subunit) and PSMA3 (19S subunit) were confirmed by immunoprecipitation and immunoblot analysis that used extracts prepared from human embryonic kidney 293 (HEK293) cells expressing GFP epitope-tagged SERBP1 (Fig. 4C).

Since the 26S proteasome has been implicated in the elimination of SGs induced by arsenite and heat stress [9,10], we tried to investigate whether SERBP1 and the 26S proteasome possessed overlapping effects on the turnover of arsenite-induced SGs. To this end, we examined the impact of the 26S proteasome inhibitor bortezomib (BTZ) on SG clearance in control and SERBP1 KD HeLa cells. We treated cells with a low dose of BTZ and found that BTZ can inhibit the proteasome but does not induce SG formation. Interestingly, treatment with BTZ simultaneously with arsenite stress induced almost complete blockage of SG elimination after the 2-h recovery phase in control cells (Fig. 4D and E), indicating that the 26S proteasome plays a dominant role during SG disassembly. However, adding BTZ to SERBP1 KD cells had similar effects on SG disassociation to those in control cells, suggesting that SERBP1 potentially acts as an upstream modulator or functions in parallel to the 26S proteasome (Fig. 4D and E).

The inhibitory effect of BTZ on SG clearance indicated the possible requirement of 26S proteasome presence or activation at SGs. We thus examined 20S (catalytic subunit of 26S) proteasome activity in the context of SG induction and disassociation and investigated whether SERBP1 was involved in this process. The 20S proteasome activity was evaluated by measuring 7-amino-4-methyl coumarin (AMC) released from the commercial fluorogenic peptide substrate Suc-LLVY-AMC that can be hydrolyzed by the proteasome in cell extracts. Inhibition of proteasome activity by BTZ was tested in parallel as a negative control, which confirmed the reliability of this assay. Under unperturbed conditions, the basal level of proteasome activity was comparable in the presence or absence of SERBP1 (Fig. S4A), whereas overexpression of SERBP1 induced slightly increased proteasome activity, which can be explained by the fact that SERBP1 overexpression triggered weak SG formation (Fig. S4B). Intriguingly, we observed a significantly reduced AMC fluorescence signal in SERBP1 KD cells during the recovery phase from arsenite stress, suggesting that SERBP1 could manipulate proteasome activation (Fig. S4C). Therefore, we tried to determine whether proteasome activity was correlated with SG formation and disassociation and whether SERBP1 could contribute to this correlation. The proteasome activities of protein were examined in cell extracts immediately after exposure to arsenite stress and after being permitted to recover for 1 to 2 h and were simultaneously compared with those in the untreated control. A noticeable elevation of AMC fluorescence intensity can be detected in control cells with arsenite stress treatment compared with the untreated control cells, and a rapidly declining signal in the subsequent recovery phase after arsenite removal was observed, which displayed wavelike changes with the addition and withdrawal of arsenite stress (Fig. 4F). Conversely, in the SERBP1 KD cells, a distinct dynamic pattern of AMC signal was observed among the corresponding situations, indicating an impaired proteasome activity-responsive mechanism upon SERBP1 deficiency (Fig. 4F). However, the protein levels of proteasome composition factors such as PSMA3 (20S) and PSMD10 (19S) were not changed in SERBP1 KD cells compared with WT control cells (Fig. S4D), which suggests that SERBP1 does not manipulate proteasome activity dynamics by affecting the protein levels of proteasome subunits. We then measured the levels of proteasome activity in SERBP1 KD HeLa cells ectopically expressing WT SERBP1 to further confirm that SERBP1 could promote proteasome activation under arsenite stress. As expected, reintroducing SERBP1 restored the ability of cells to activate the proteasome to comparable levels in control cells in response to arsenite stress, whereas BTZ eliminated the differences among the tested groups (Fig. 4G and Fig. S4E).

Furthermore, we characterized the dynamics of proteasome activity during the heat shock and recovery stages. Similarly, in control cells, a markedly enhanced AMC signal was observed after heat shock exposure and subsequently declined after moving cells to the normal culture state. In SERBP1 KD cells, although we also detected a dynamic change in cells from the untreated, heat shock, and recovery phases, the peak values were strongly reduced compared with the corresponding conditions in control cells, indicating that the proteasome activation pathway was impaired upon SERBP1 depletion (Fig. 4H and Fig. S4F). In addition, complementation experiments further revealed that ectopic expression of SERBP1 fully rescued the impaired proteasome activity in SERBP1 KD cells (Fig. 4I), thereby formally proving that the defective activation was due to the lack of SERBP1. Additionally, we observed comparable levels after proteasome inhibitor BTZ treatment, supporting the reliability of this experiment (Fig. 4I and Fig. S4G). Together, these data indicate that intact proteasome activity was indispensable for efficient SG clearance and underscored that the compromised proteasome activity due to SERBP1 depletion was linked to defective SG clearance. In addition, these results further indicate that SERBP1 acts as an upstream modulator of proteasome activation in SG turnover.

### SERBP1 mediates recruitment of the 26S proteasome and VCP into SGs

**Fig. 5.**
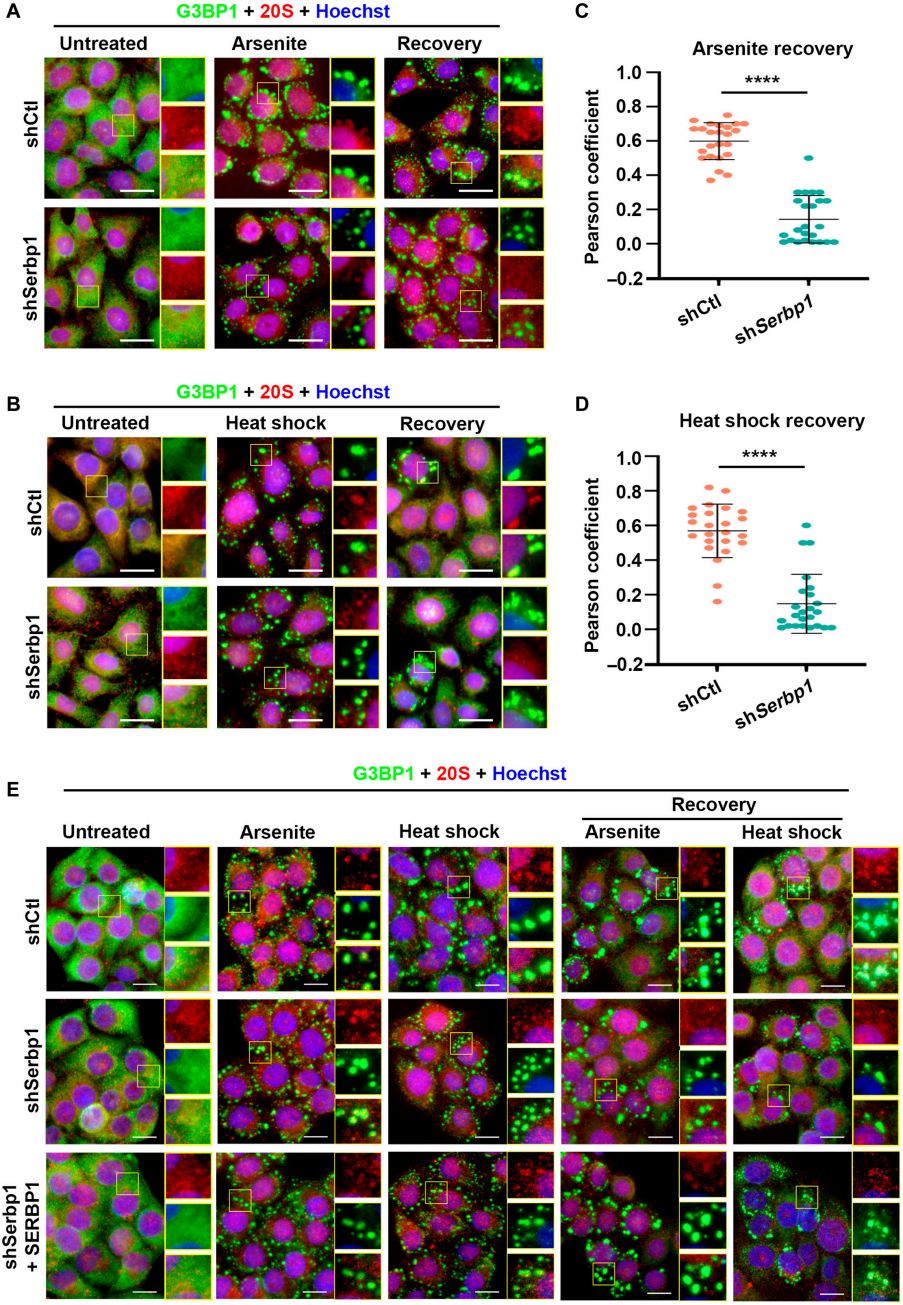
SERBP1 mediates 20S proteasomal localization in SGs during the disassembly process. (A and B) HeLa cells stably depleted SERBP1 (shSERBP1) using shRNA lentivirus or HeLa cells with control shRNA (shCtl), were treated with 0.5 mM sodium arsenite for 60 min (Arsenite), and then allowed to recover for 60 min (Recovery) (A), or cultured at 43 °C for 60 min (Heat shock) and then allowed to recover for 30 min (Recovery) (B). 20S and SG marker G3BP1 were detected by immunofluorescence and visualized by confocal microscopy. Scale bars = 10 μm. (C and D) Scatterplot representing the colocalization correlation between G3BP1 and 20S signal during recovery stage from AS (C) or heat shock (D). Data are shown as means ± SD. Statistical significance was determined using an unpaired *t* test comparison. Each dot represents the Pearson coefficient analysis of a single ROI. Three experiments were performed independently. ****P < 0.0001. (E) HeLa cells stably depleted SERBP1 (shSERBP1) using shRNA lentivirus or SERBP1 depleted HeLa cells transfected with plasmid encoding SERBP1, or HeLa cells with control shRNA (shCtl) were either treated with 0.5 mM arsenite for 60 min (Arsenite), and allowed to recover for 120 min after drugs removal (Arsenite Recovery), or cultured at 43 °C for 1 h (Heat shock), and allowed to recover for 30 min (37 °C, Heat shock Recovery), and then 20S and SG marker G3BP1 were detected by immunofluorescence and visualized by confocal microscopy. Scale bars = 10 μm.

It has been reported that the 20S and 19S subunits of the 26S proteasome can colocalize into SGs, which is required for efficient SG clearance and turnover [8,10]. Based on our findings that SERBP1 involves diverse stressor-induced SGs and interacts with multiple 26S proteasome components, we assumed that SERBP1 might mediate the recruitment of proteasome subunits in addition to its effect on proteasome activity. To investigate whether SERBP1 regulates endogenous 26S proteasome recruitment, we analyzed the localization of the 26S proteasome upon arsenite and heat shock stress using a 20S-specific antibody in WT control cells and SERBP1 KD cells. The results showed that hardly any colocalization of 20S and G3BP1 was detected in either control or SERBP1 KD cells without arsenite or heat shock stress, whereas partial colocalization of G3BP1 and 20S in cytoplasmic aggregates was observed in control and SERBP1 KD cells, which was also observed after a 2-h recovery phase in SG-positive cells (Fig. 5A and B). Interestingly, in SERBP1 KD cells under arsenite or heat shock stress conditions, there was a substantial reduction in 20S-containing SGs compared to control cells, especially during the recovery phase (Fig. 5A to D and Fig. S5A and B). To further confirm the role of SERBP1 in 20S localization, a rescue assay was performed by transfecting SERBP1 KD HeLa cells with a plasmid encoding WT SERBP1. The results showed that reexpression of SERBP1 in SERBP1-deficient cells could fully rescue the impaired 20S localization within SGs in the recovery phase from arsenite or heat shock stress conditions (Fig. 5E and Fig. S5C), further proving that SERBP1 is required for the normal localization of the 20S subunit of the 26S proteasome during SG disassembly. We inferred from these results that SERBP1 is required to maintain the 20S subunit of the 26S proteasome within SGs during the recovery phase, thereby playing a critical role in SG disassembly.

**Fig. 6.**
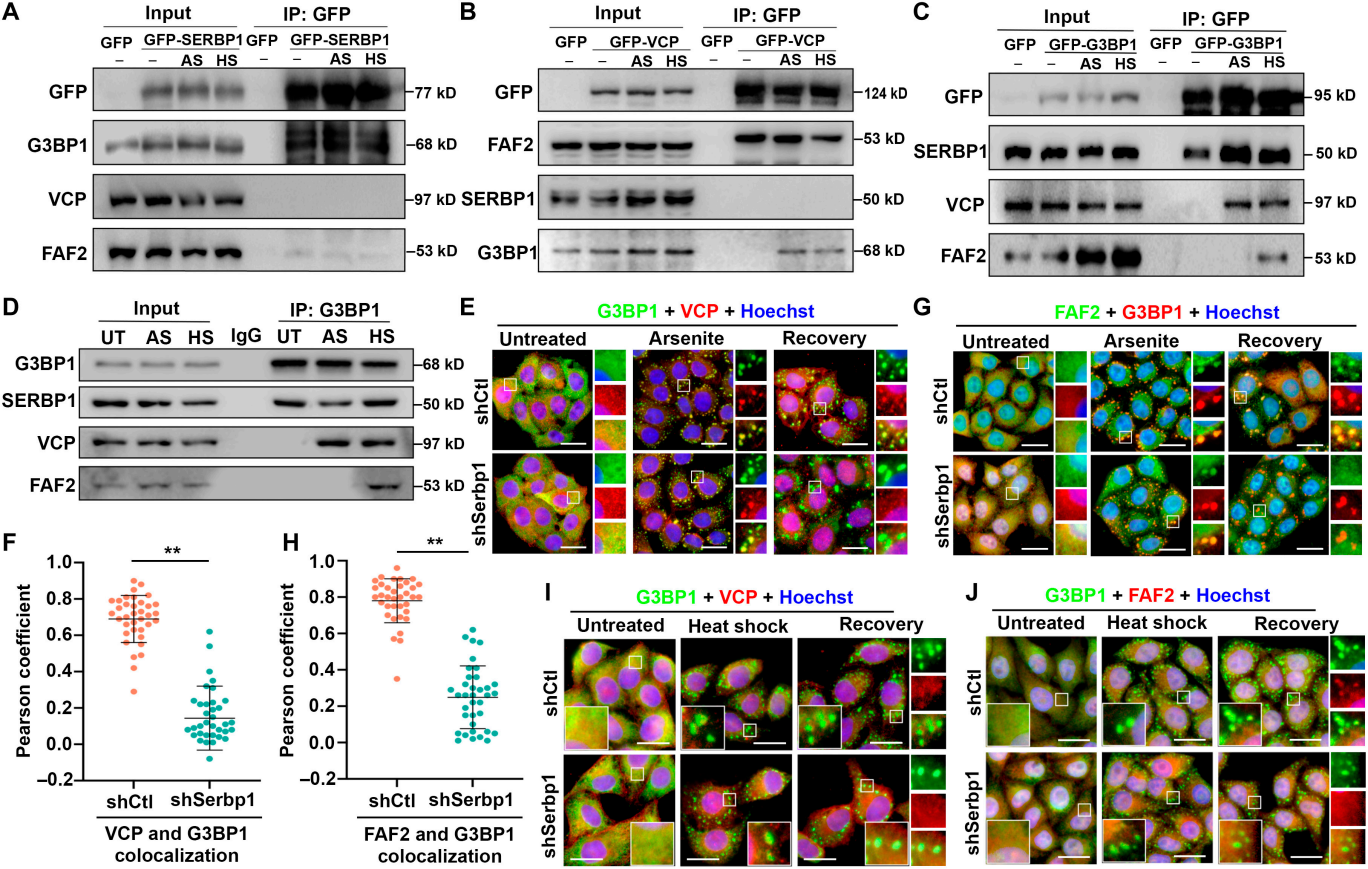
SERBP1 modulates VCP and FAF2 recruitment into SGs during the disassembly process. (A to C) Images of Western blot of lysates from the co-IP experiment are shown. HEK293 cells expressing GFP-tagged SERBP1 (A), GFP-tagged VCP (B), and GFP-tagged G3BP1 (C), respectively, were treated with 0.5 mM sodium arsenite for 60 min (AS) or cultured at 43 °C for 1 h (HS) and subjected to anti-GFP IP. Input and IP samples were immunoblotted for indicated proteins. (D) Images of the Western blot of lysates from the co-IP experiment are shown. HeLa cells were treated with 0.5 mM sodium arsenite (AS) for 60 min or cultured at 43 °C for 1 h (HS) or untreated with any stress (UT) and then subjected to anti-G3BP1 IP. The membrane was immunoblotted with G3BP1, SERBP1, VCP, and FAF2 antibodies to detect protein levels in input and IP samples. (E) HeLa cells stably depleted SERBP1 (shSERBP1) using shRNA lentivirus or HeLa cells with control shRNA (shCtl), were treated with 0.5 mM sodium arsenite for 60 min (Arsenite), and then allowed to recover for 60 min (Recovery). VCP (red) and SG marker G3BP1 (green) were detected by immunofluorescence and visualized by confocal microscopy. Scale bars = 10 μm. (F) Scatterplot representing the colocalization correlation between G3BP1 and VCP signal during recovery stage in (E). Data are shown as means ± SD. Statistical significance was determined using an unpaired *t* test comparison. Each dot represents the Pearson coefficient analysis of a single ROI. Three experiments were performed independently. **P < 0.01. (G) HeLa cells stably depleted SERBP1 (shSERBP1) using shRNA lentivirus or HeLa cells with control shRNA (shCTL) were treated with 0.5 mM sodium arsenite for 60 min (Arsenite) and then allowed to recover for 60 min (Recovery). FAF2 (green) and SG marker G3BP1 (red) were detected by immunofluorescence and visualized by confocal microscopy. Scale bars = 10 μm. (H) Scatterplot representing the co-localization correlation between FAF2 and G3BP1 signal during recovery stage in (G). Data are shown as means ± SD. Statistical significance was determined using an unpaired *t* test comparison. Each dot represents the Pearson coefficient analysis of a single ROI. Three experiments were performed independently. **P < 0.01. (I and J) HeLa cells stably depleted SERBP1 (shSERBP1) using shRNA lentivirus or HeLa cells with control shRNA (shCtl), were cultured at 43 °C for 1 h (Heat shock) and allowed to recover for 60 min (Recovery). Co-staining of VCP (I) and FAF2 (J) with SG marker G3BP1 was detected by immunofluorescence and visualized by confocal microscopy. Scale bars = 10 μm.

We next pursued the finding from our proteomics studies that SERBP1 interacts with VCP (Table S1), a ubiquitin-selective AAA-adenosine triphosphatase that is best known as a regulator of SG degradation [11]. However, in contrast to the proteomics data, no detectable VCP signal was observed in the co-immunoprecipitation (co-IP) experiments by ectopically expressing GFP-SERBP1 in HEK293T cells (Fig. 6A). Moreover, reciprocal co-IP assays revealed that GFP-VCP expressed in HEK293T cells could not pull down SERBP1 (Fig. 6B). These results indicated a weak and/or indirect interaction between the VCP and SERBP1 proteins. Since the SG nucleator protein G3BP1 showed specific coprecipitation with SERBP1 and VCP (Fig. 6A and B) and VCP has been reported to interact with G3BP1 in mediating SG disassembly [11], we speculate that G3BP1 could be the core factor linking SERBP1 and VCP as well as FAF2 to form a functional complex. To test this hypothesis, co-IP was performed in lysates from HEK293T cells expressing GFP-G3BP1. Clearly, both SERBP1 and VCP exhibited G3BP1 binding activity in either arsenite-treated or heat shock-treated cells (Fig. 6C). Consistently, a co-IP assay using a G3BP1 antibody enabled us to detect the co-IP of endogenous G3BP1 with SERBP1 and VCP in arsenite-treated or heat shock-treated cells (Fig. 6D), supporting the notion that G3BP1 is the core mediator linking SERBP1 and VCP.

Given that VCP accumulates in both arsenite- and heat shock-induced SGs [10,14], we investigated whether its localization in SGs at the different assembly and disassembly stages is SERBP1 dependent. Upon arsenite stress, strong and almost complete colocalization of VCP and G3BP1 in cytoplasmic aggregates was observed in both control and SERBP1 KD cells (Fig. 6E), indicating the successful recruitment of VCP during SG assembly. However, during the recovery stage, the colocalization of VCP and G3BP1 within undissociated SGs was greatly disrupted in SERBP1 KD cells (Fig. 6E and F). Subsequently, to further strengthen the evidence of mislocalization of VCP, we tested whether the localization of FAF2, which engaged VCP for SG clearance, was also affected. As expected, SERBP1 KD did not influence FAF2 accumulation in SGs in arsenite-treated cells; however, a strongly reduced FAF2 and G3BP1 colocalization coefficient was observed in SERBP1 KD cells compared to control cells during the recovery stage (Fig. 6G and H). Moreover, in line with the observed impairments of VCP and FAF2 localization in arsenite-induced SGs, we observed that their maintenance within heat shock-induced SGs in the disassembly stage was also explicitly affected by SERBP1 depletion (Fig. I and J). Together, these data underscore that VCP and FAF2 are associated with SG assembly and that the SERBP1-mediated persistence of VCP and FAF2 within SGs is required for their efficient clearance.

### SERBP1 is indispensable for G3BP1 polyubiquitination during heat shock-induced SG clearance

**Fig. 7.**
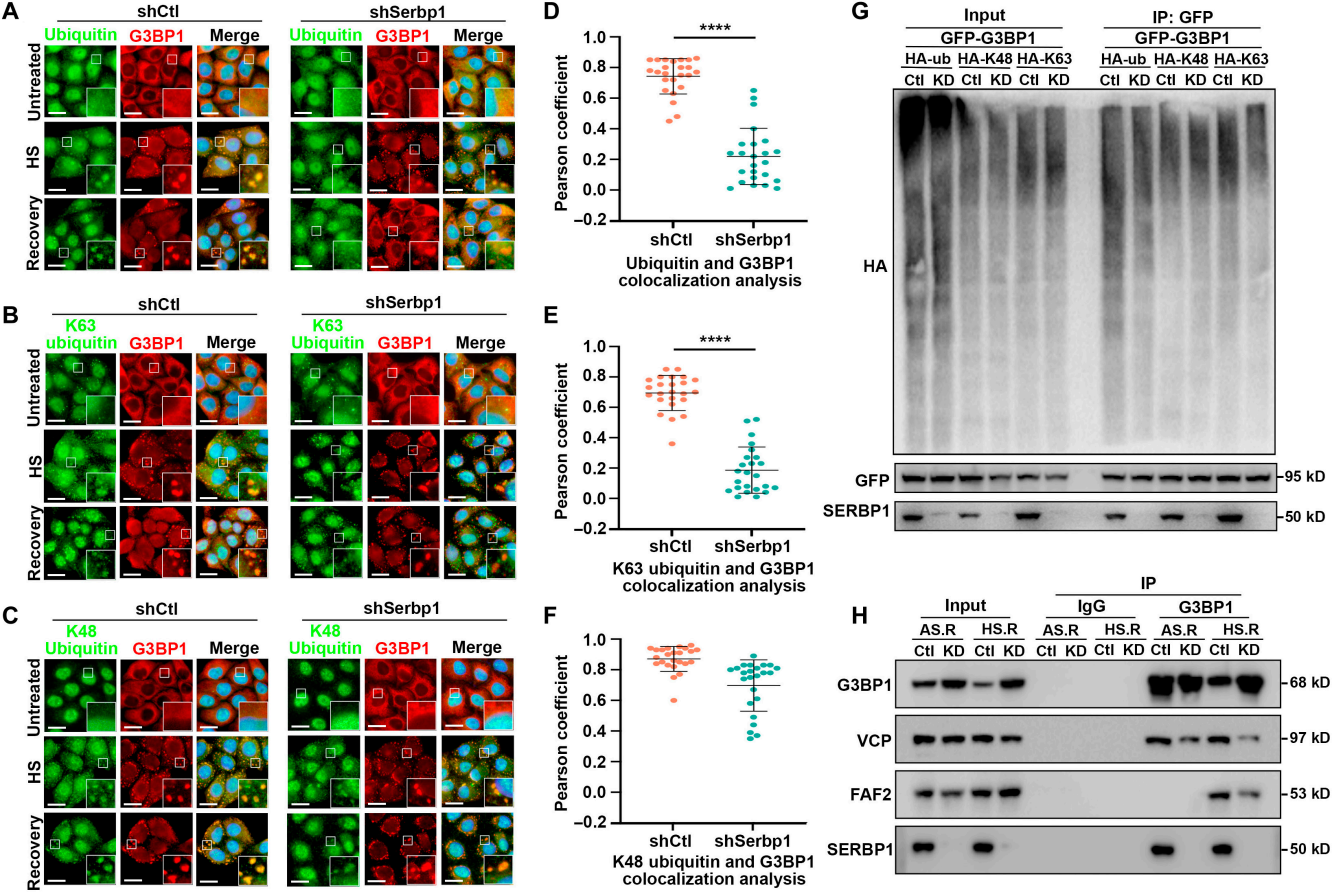
SERBP1 depletion affects G3BP1 ubiquitination level in response to heat shock. (A to C) HeLa cells stably depleted SERBP1 (shSERBP1) using shRNA lentivirus or HeLa cells with control shRNA (shCtl), were at 43 °C for 1 h (Heat shock) and allowed to recover for 60 min. Pan protein ubiquitination (A), K63-linked protein ubiquitination (B), or K48-linked protein ubiquitination (C) and SG marker G3BP1 were detected by immunofluorescence and visualized by confocal microscopy. Scale bars = 10 μm. (D to F) Scatterplot representing the colocalization correlation between G3BP1 and Pan ubiquitination signal (D), K63-linked ubiquitination signal (E), and K48-linked ubiquitination signal (F) during recovery stage. Data are shown as means ± SD. Statistical significance was determined using an unpaired *t* test comparison. Each dot represents the Pearson coefficient analysis of a single ROI. Three experiments were performed independently. ****P < 0.0001. (G) Immunoblots of cell extracts immunoprecipitated with GFP antibody showing G3BP1 ubiquitination. HEK293T cells were transfected with either SERBP1 shRNA or control shRNA; 48 h after transfection, cells were cotransfected with GFP-G3BP1 and ubiquitin (HA-ub, HA-K48, and HA-K63), and cultured at 43 °C for 1 h and recovery (37 °C, 30 min) and then subjected to anti-G3BP1 IP. The membrane was immunoblotted with HA, GFP, and SERBP1 antibodies to detect protein levels in input and IP samples. K48 and K63 permit K48-linked or K63-linked chains exclusively. HA, hemagglutinin. (H) Images of Western blot of lysates from the co-IP experiment are shown. HeLa cells stably depleted SERBP1 (shSERBP1) using shRNA lentivirus (KD) or HeLa cells with control shRNA (Ctl) were treated with 0.5 mM sodium arsenite for 1 h and recovered for 1 h (AS R) or cultured at 43 °C for 1 h and recovered for 30 min at 43 °C (HS R), respectively, then subjected to anti-G3BP1 IP. The membrane was immunoblotted with G3BP1, VCP, FAF2, and SERBP1 antibodies to detect protein levels in input and IP samples.

Because of the observed disappearance of FAF2 and VCP within SGs during the recovery phase, we reasoned that SERBP1 could contribute to SG disassembly via G3BP1-FAF2/VCP ubiquitination-dependent pathways. To explore this possibility, we examined the signal of panubiquitin and 2 functionally well-defined polyubiquitin chain linkage types (K48- and K63-linked ubiquitination) within heat shock-induced SGs using linkage-specific antibodies. Under basal conditions, all types of ubiquitin signals were uniformly distributed, whereas they readily and robustly accumulated in SGs upon heat shock in both control and SERBP1 KD cells (Fig. 7A to C). Interestingly, we found that in SERBP1 KD cells, a significant decrease in panubiquitin and K63-linked polyubiquitin signals was observed in SGs after 1 h of recovery compared to those observed in control cells (Fig. 7D and E). In contrast, the colocalization of the K48-linked polyubiquitin signal with G3BP1 did not change in SGs upon SERBP1 knockdown (Fig. 7F). These data suggest that SERBP1 promotes SG disassembly after heat shock stress through panubiquitin and K63-linked polyubiquitin, not the K48-linked polyubiquitin pathway.

G3BP1 is one of the key substrates that undergoes K63-linked polyubiquitination in response to heat shock stress [13]. To further determine whether SERBP1 is required for K63-linked polyubiquitination of G3BP1 during SG clearance, we performed a ubiquitination assay in SERBP1 KD cells under heat shock stress. We used ubiquitin mutants that permit only exclusively K48-linked or K63-linked chains (in which the only available lysine is K48 or K63) to distinguish these 2 dominant polyubiquitination linkage types. The results showed that expression of WT or K63 ubiquitin in SERBP1 KD cells inhibited the accumulation of polyubiquitinconjugated G3BP1 during the recovery phase, whereas expression of K48 ubiquitin had no impact (Fig. 7G), which further proves that SERBP1 promotes heat shock-induced K63-linked polyubiquitination of G3BP1. Since FAF2 is the adaptor linking G3BP1 and VCP in a ubiquitin-dependent manner that is indispensable for mediating the disassembly of heat shock-induced SGs, we hypothesized that the regulation of G3BP1 polyubiquitination by SERBP1 might be dependent on the G3BP1 interaction with FAF2 and VCP during SG clearance. Indeed, we detected an apparent reduction in endogenous FAF2 and VCP co-immunoprecipitated by G3BP1 antibody in SERBP1 KD cells during the recovery phase, confirming the importance of SERBP1 in regulating the G3BP1-FAF2/VCP polyubiquitination-dependent pathway (Fig. 7H). Taken together, these results reveal that SERBP1 is indispensable for G3BP1 polyubiquitination events and might promote SG clearance by regulating the ubiquitinated G3BP1-FAF2/VCP axis in these contexts.

### SERBP1 protects male germ cells in mouse testes from thermal damage in vivo and is essential for spermatogenesis

Spermatogenesis in most mammals is a highly thermosensitive process and therefore occurs in testes maintained at a lower temperature than the core body [24]. SGs can be detected in male germ cells upon heat shock stress and function as a protective mechanism against apoptosis from thermostimuli damage [16]. The implications of SERBP1 in SG dynamics prompted us to hypothesize that SERBP1 could be involved in mouse spermatogenesis by regulating stress responses in germ cells. To test this hypothesis, we sought to generate *Serbp1* KO mice and determine the functional significance of SERBP1 in vivo*.* Unfortunately, none of the *Serbp1* KO mice were born from the intercross of heterozygotes, which indicated the possibility of embryonic lethality induced by *Serbp1* deficiency. We observed typical genotypes of WT, heterozygote, and KO embryos at embryonic day 14.5 (E14.5) from an intercross of heterozygotes and verified them by Western blotting with a specific SERBP1 antibody (Fig. S6A and B). Furthermore, we collected and genotyped embryos from different embryonic days to dissect the timeline of KO embryo loss. The morphological abnormality of KO embryos appeared as early as E10.5, and death occurred by E17.5, with markedly increased lethality after E14.5 (Fig. S6C and D). Interestingly, we noted that the cell numbers of KO fetal livers were significantly less than those in WT littermates, and the size was smaller than that of WT littermates at E14.5, which suggested that *Serbp1* deletion might result in hematopoietic defects (Fig. S6C and D).

**Fig. 8.**
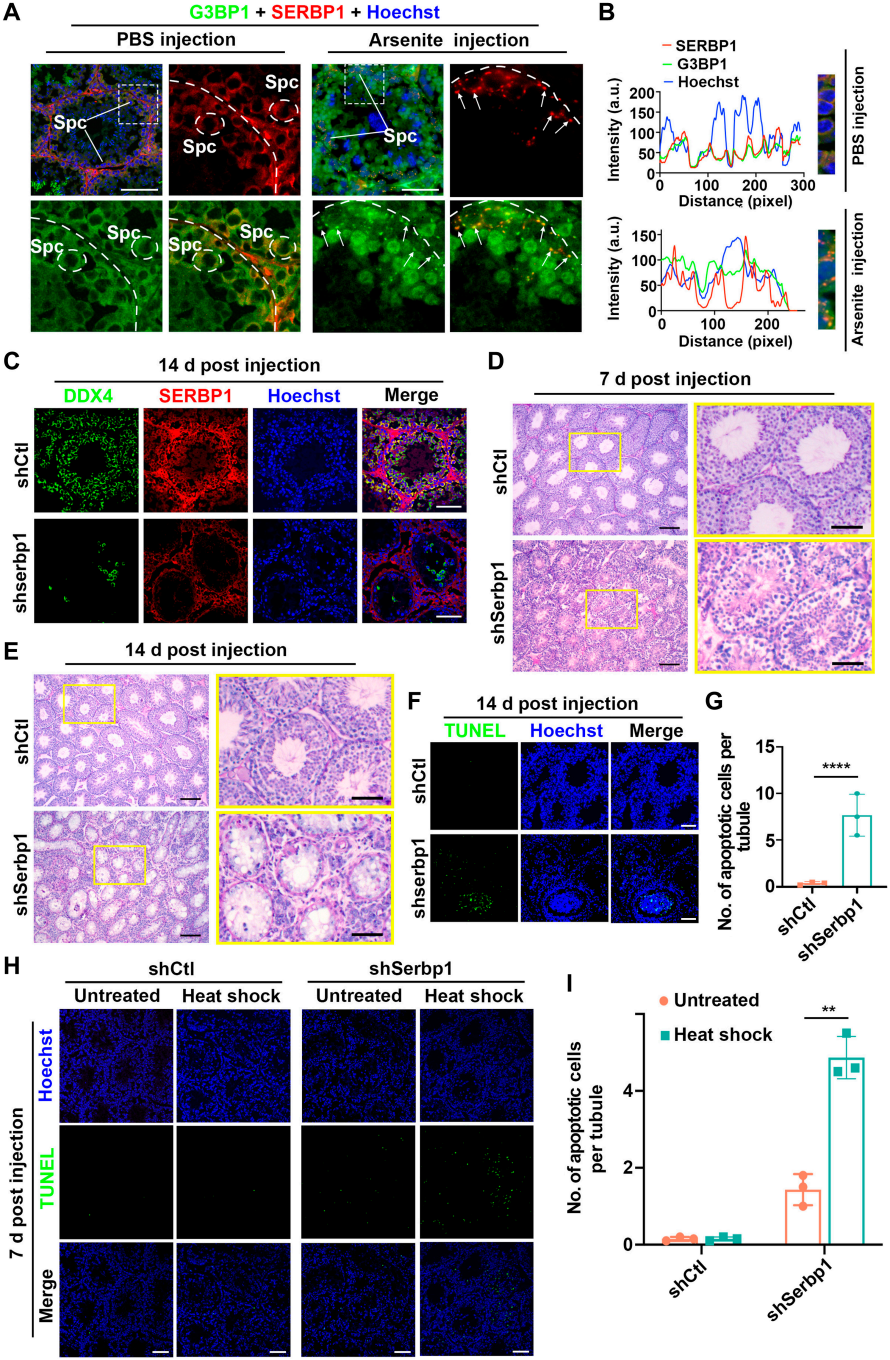
SERBP1 colocalizes with SG marker G3BP1 in testis and protects germ cells from apoptosis after heat shock. (A) Representative immunofluorescence images of costaining SERBP1 with the general SG marker G3BP1 on mouse testicular sections are shown. Localization of SERBP1 and G3BP1 in WT mouse testes injected either with PBS or sodium arsenite. Mouse testes were injected either with PBS or sodium arsenite via the efferent ducts. Two hours post-injection, testes were collected and cryosections were made accordingly. The dotted cycles indicate spermatocytes. Scale bars = 50 μm. (B) The signal profiles of the ROI from (A) to analyze colocalization of G3BP1 and SERBP1 in PBS injection and arsenite injection groups, respectively, using ImageJ software. The images represent the magnification of the ROI with the merged channel. The graph corresponds to the normalized pixel-by-pixel gray value of the 2 images. (C) Representative images showing staining for DDX4 and SERBP1 on the control and SERBP1 KD mouse testis sections. Mouse testes were injected with either control shRNA or SERBP1 shRNA lentivirus via the efferent ducts. Fourteen days post-injection, testes were collected and cryosections were made accordingly. Scale bars = 50 μm. (D and E) Histological analysis of control or SERBP1 knockdown testes. Mouse testes were injected with either control shRNA or SERBP1 shRNA lentivirus via the efferent ducts. Seven (D) or 14 d (E) post-injection, testes were collected and periodic acid-Schiff staining was performed. Scale bars = 50 μm. (F) Cell apoptosis detected by TUNEL assay. Mouse testes were injected with either control shRNA or SERBP1 shRNA lentivirus via the efferent ducts. Fourteen days post-injection, testes were collected and cryosections were made accordingly. Scale bars = 50 μm. (G) Quantification of apoptotic cells in control and SERBP1 knockdown mouse testes for (F). Three mice were used to analyze for each condition. Data are shown as means ± SD. Statistical significance was determined using an unpaired *t* test comparison. ****P < 0.0001. (H) Cell apoptosis detected by TUNEL in control or SERBP1 knockdown testes in response to heat shock. Mouse testes were injected with either control shRNA or SERBP1 shRNA lentivirus via the efferent ducts. Fourteen days post-injection, mice were heat stressed (43 °C, 2 h) and allowed to recover for 2 h. Testes were collected and cryosections were made accordingly. Scale bars = 50 μm. (I) Quantification of apoptotic cells in control and SERBP1 knockdown mouse testes for (H). Three mice were used to analyze for each condition. Data are shown as means ± SD. Statistical significance was determined using an unpaired *t* test comparison. **P < 0.01. a.u., arbitrary units.

Because of the lack of a powerful KO mouse model, we attempted to evaluate the significance of SERBP1 for the male germ cell stress response. First, we subjected mouse testes to arsenite injection to establish a short-term and acute SG induction model in vivo. By coimmunostaining the common SG marker TIAR with the germ cell-specific SG marker DAZL, we observed that TIAR could colocalize with DAZL in the cytoplasm of spermatogonia and spermatocytes at 3 h post administration of sodium arsenite solution (Fig. S7A and B), indicating that SGs can be induced in mouse testicular cells in vivo. Notably, coimmunostaining of SERBP1 with G3BP1 in testis sections revealed that the SERBP1 foci in the cytoplasm of germ cells were nearly completely G3BP1-positive when germ cells were exposed to arsenite stress, whereas SERBP1 was evenly distributed in the cytoplasm of germ cells in testes injected with phosphate buffer saline (PBS) (Fig. 8A and B). This result suggests that SERBP1 can also be recruited into SGs in male germ cells and may play a critical role in germ cell development.

To assess the physiological role of SERBP1 in spermatogenesis and male germ cell development, we generated SERBP1 KD mouse testes (KD mice) using lentivirus transduction injected into seminiferous tubules through the efferent duct. We first verified the knockdown efficiency of lentivirus carrying specific shRNA against the mouse *Serbp1* gene in the mouse spermatogenic cell lines GC-1 and GC-2. The results showed significant depletion of the *Serbp1* gene at both the mRNA and protein levels, supporting the application of shRNA lentivirus in mouse testes (Fig. S7C to E). As expected, after injection of the concentrated virus into the testes, a decrease in SERBP1 protein levels was detected on day 7; in parallel, SERBP1 signal costaining with the germ cell marker TRA98 was also reduced, and a further persistent reduction was observed on day 14 (Fig. 8C and Fig. S7F and G). Notably, *Serbp1* KD testes exhibited loss of DDX4-labeled germ cells on both days 7 and 14, whereas there was no detectable impact on the WT1-labeled Sertoli cells, indicating that impaired spermatogenesis or germ cell survival occurred in *Serbp1* KD testes (Fig. 8C and Fig. S7H and I). Further histological analysis of seminiferous tubules from control and *Serbp1* KD testes revealed disorganized layers of spermatogenic cells at 7 d after virus injection and an increased number of damaged seminiferous tubules with vacuoles and lost germ cells at day 14, indicating a crucial role of *Serbp1* in spermatogenesis and male germ cell development (Fig. 8D and E). Consistently, a significantly increased cell population undergoing apoptosis was observed 7 d after virus injection, as evidenced by aggregation of the apoptotic signal in *Serbp1* KD seminiferous tubules by TUNEL (Terminal Deoxynucleotidyl Transferase mediated dUTP Nick-End Labeling) assay (Fig. 8F and G). Interestingly, we found that the number of apoptotic germ cells was significantly increased in *Serbp1* KD testes compared with control testes upon scrotal heat shock stress (Fig. 8H and I), suggesting that SERBP1 could protect male germ cells in mouse testes from thermostimuli damage. Taken together, these in vivo data infer that SERBP1 is involved in SG dynamics during spermatogenesis and contributes to its protection of thermosensitive male germ cells from apoptosis against thermostimuli.

## Discussion

**Fig. 9.**
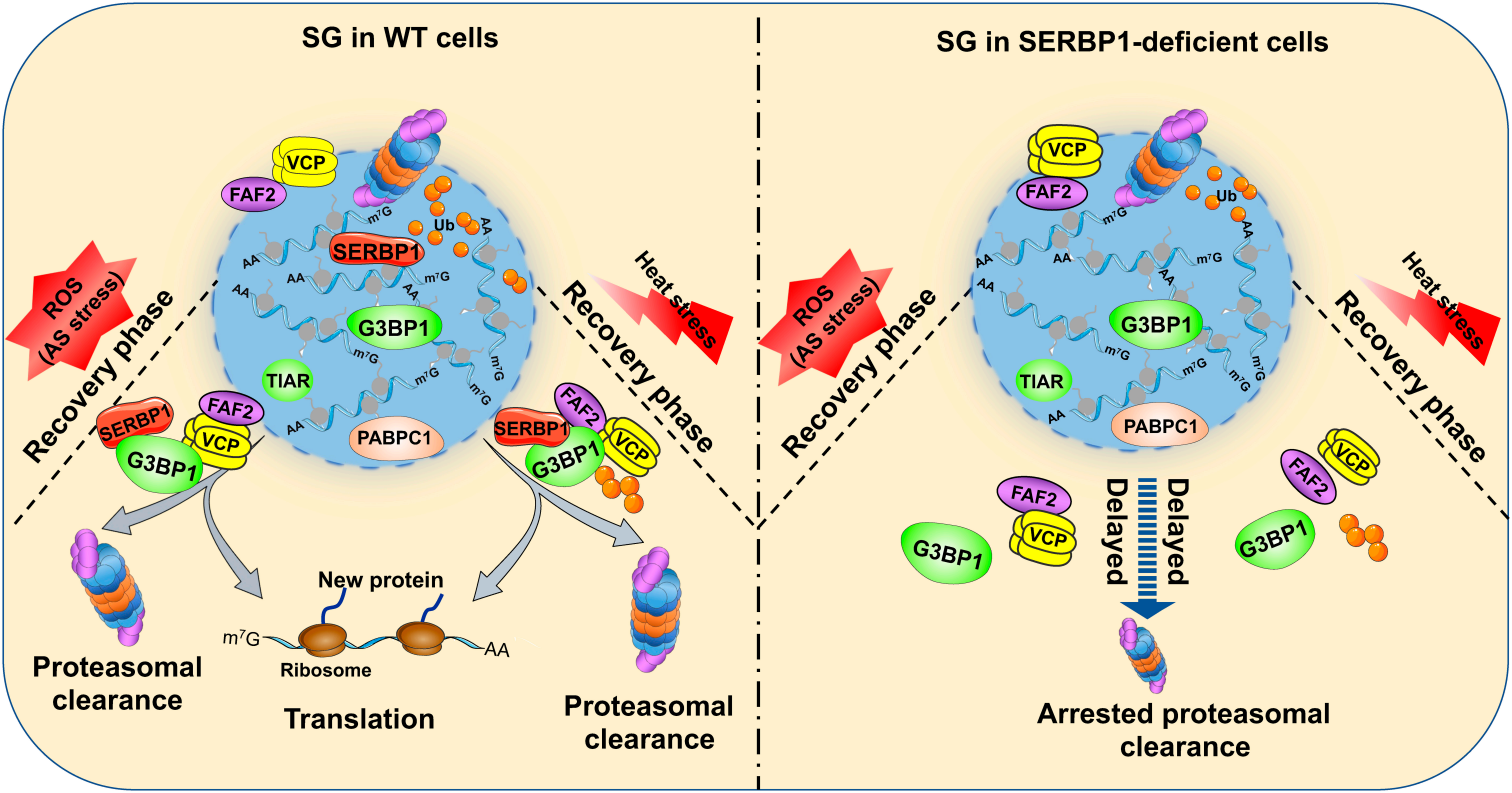
The schematic illustration shows the working model of SERBP1 in regulating SG clearance in cells during the recovery phase. ROS, reactive oxygen species.

In the current study, we identified SERBP1 as a universal SG-associated factor that acted as a critical regulator of arsenite- and heat shock-induced SG elimination and demonstrated that SERBP1 exhibited a protective effect on germ cell survival against heat shock stress. The role of SERBP1 in SG metabolism can be summarized as follows: (a) Arsenite induces the formation of SGs consisting of stalled translation initiation complexes, untranslated mRNAs, RNA-binding proteins with low-complexity regions, such as TIA-1, G3BP1, and PABPC1, and dozens of additional proteins contributing to SG structure and dynamics, including VCP and FAF2. (b) SERBP1 mediates the recruitment of P97 and the 26S proteasome and promotes proteasome activity in various stimuli-induced SGs, thereby facilitating SG clearance. (ii) Specifically, in the context of heat shock, SERBP1 depletion reduces the localization of K63-linked ubiquitinated proteins in SGs and impairs the G3BP1 ubiquitination level during the recovery process, thus weakening the interaction with VCP and FAF2, which is the critical molecular event for SG disassembly (Fig. 9).

The growing list of SG-associated proteins has been investigated using powerful proteomics approaches in the past few years [3,25]. In this study, although SERBP1 has been reported to colocalize with the SG marker TIA-1 upon arsenite treatment, as observed by immunofluorescence [23], we noticed that SERBP1 was not listed as a mammalian cell SG component by any of the previous proteome studies characterizing SG-related proteins. This biased result may be due to noncomprehensive lists; some components may be lost during the purification process, poorly detected, or unique to specific stress conditions. Therefore, with our coimmunostaining of SERBP1 with G3BP1 in the current study, we clarified that SERBP1 could be recruited into SGs induced by various stressors, making it a common SG-associated factor (Fig. 1A and B). A critical characteristic of bona fide SGs is the reversibility after stress removal or upon cotreatment with the translation elongation inhibitor cycloheximide. SERBP1 diffused in the cytoplasm upon the addition of cycloheximide, further suggesting that the SERBP1 puncta that appear under stress conditions are indeed SGs. Interestingly, we observed spontaneous G3BP1 foci in SERBP1-GFP-expressing cells (both in WT and mutants), even under unperturbed conditions. The possible explanation for induced SGs is likely that SERBP1 can inactivate ribosomes by occupying the ribosomal mRNA entrance channel, resulting in translational repression of specific ribosomes [26].

Furthermore, posttranslational modifications of proteins, such as the arginine methylation of UBAP2L [27], the phosphorylation of growth factor receptor-bound protein 7 (Grb7) [28], the ubiquitination of G3BP1 [13], the SUMOylation pathway [29], and PARylation [30], have been reported to contribute substantially to SG disassembly. In contrast, the implications of RNA binding proteins during SG clearance are less addressed. In the current study, one of the key findings is our identification of SERBP1, which promotes SG clearance under both arsenite stress and heat shock conditions. This finding expanded the understanding of the molecular mechanism underlying SG disassembly. More importantly, the SERBP1 interactome revealed the surprising finding that it associates with a set of subunits of the 26S proteasome, prompting us to dissect the potential link mediated by SERBP1 for SG clearance. Given that the 26S proteasome is directly involved in the clearance of arsenite-induced SGs and that impaired proteasome function causes transformation into aberrant SGs [10], the effect of SERBP1 on the proteasome was assessed in this study. Intriguingly, we obtained the exciting finding that 26S proteasome activity dynamically changed with SG assembly and disassembly processes, and notably, SERBP1 specifically affects proteasome activity in cells that have recovered from the stressors. This phenomenon can be observed in both arsenite stress and heat shock, suggesting that it may be a common shared mechanism of SG clearance induced by these 2 different conditions. In addition, SERBP1 loss of function impaired 20S proteasome recruitment during recovery from either arsenite stress or heat shock conditions, in contrast to ZFAND1, which specifically modulates 20S recruitment to eliminate arsenite-induced SGs [10].

Given their well-established and crucial roles in SG elimination, VCP and FAF are other SERBP1 interactors of interest. However, we did not detect their interaction with each other using a co-IP experiment in this study, which is inconsistent with the mass spectrometry data, indicating a weak or indirect interaction between SERBP1 and VCP/FAF. Indeed, further co-IP analysis showed that the SG core protein G3BP1 could co-immunoprecipitate VCP and SERBP1 upon arsenite stress and heat shock and interact with FAF2 specifically under heat shock stress, indicating that G3BP1 may be a mediator linking SERBP1 and either VCP or FAF2 in a stress-dependent manner. A previous study revealed that the recruitment of VCP and FAF2 is a prerequisite for SG removal, while the mislocation of VCP during the recovery stage caused by depletion of its regulators, such as ZFAND1, leads to inefficient SG clearance [10]. Interestingly, our study showed that SERBP1 modulates VCP and FAF2 recruitment in response to both arsenite stress and heat shock stress, whereas ZFAND1 only affects VCP localization under arsenite stress, indicating that distinct mechanisms exist for VCP recruitment by different upstream factors. Recently, a breakthrough in research on SG clearance revealed the precise molecular basis underlying SG dynamics: ubiquitylation events are critical for the disassembly of SGs formed after heat shock stress, but not for those induced by arsenite, and the ubiquitinated G3BP1-FAF2/VCP axis is specifically required for SG clearance in the setting of heat shock stress [13]. Therefore, we proposed the involvement of SERBP1 in G3BP1 polyubiquitination because we observed a reduction in K63-linked ubiquitinated G3BP1 in SERBP1 KD cells and subsequently decreased binding with VCP and FAF2, which is a reasonable explanation for the defective SG clearance.

Of note, spermatogenesis is a highly thermosensitive process requiring the maintenance and homeostasis of male germ cells for reproduction. A previous study has shown that lower temperatures are critical for normal human spermatogenesis because substantial loss of germ cells has been found in cryptorchidism and heat shock-treated testes [31]. Specifically, it has been reported that perturbed dynamics of SG in spermatogonia cause cell damage in response to heat stress by repressing the NEDD4-mediated endosomal-lysosomal pathway for SG clearance during the recovery period [32]. In the present study, we found that SERBP1 facilitates rapid SG clearance in HeLa cells by regulating G3BP1 ubiquitination and proteasome activity, which adds another possible layer of the molecular mechanism for SG disassembly during the recovery phase. Moreover, consistent with HeLa cells, SERBP1 could colocalize with G3BP1 in the cytoplasm of spermatogenic cells upon arsenite and heat shock stress, indicating that SERBP1 is linked to the physiological function of SGs in spermatogenic cells as well in response to stimuli stress. This is further supported by the fact that apoptotic germ cells were significantly increased in *Serbp1* KD testes compared with control testes upon scrotal heat shock stress. Therefore, our study reveals a possible physiological function of the RNA binding protein SERBP1 in the SG dynamics of spermatogenic cells to protect male germ cells against thermostimuli stress. Similar to this study, MSI-1, as an mRNA-binding protein, was reported to regulate the fate of Sertoli cells after heat shock-induced injury and play an essential role in supporting spermatogenesis [33]. However, further molecular insights and functional study of SG dynamics in male germ cells and the reproductive field need to be deeply explored in the future. In addition to the essential function of SERBP1 in germ cell protection against thermostimuli stress, we demonstrated its indispensable role in embryonic development. The significantly smaller fetal liver size and decreased cell number of the fetal liver in SERBP1 KO embryos suggested the possibility that the embryonic lethality may be due to hematopoietic defects. Currently, however, we cannot provide direct evidence to support this hypothesis, and more effort will be devoted to dissecting this intriguing biological function of SERBP1.

In conclusion, our data provide evidence of an SG clearance pathway mediated by SERBP1 that regulates 26S proteasome activity and G3BP1 ubiquitination and modulates SG disassociation factors, including the 20S proteasome, VCP, and FAF2. This mechanism may provide insights into reproductive research on spermatogenic cells and/or into the pathogenesis of stress-induced diseases.

## Materials and Methods

### Mice

All mice used in this study were of C57BL/6J background, and the experimental animal procedures were approved by the Institutional Animal Care and Use Committee of Tongji Medical College, Huazhong University of Science and Technology. All mice were housed in the specific pathogen-free facility of the Laboratory of Animal Center, Huazhong University of Science and Technology.

### Cell culture, plasmids, and transfection

HeLa, GC-1, GC-2, and HEK293T cells were cultured in Dulbecco’s modified Eagle medium (Gibco, 8121298) supplemented with 10% fetal bovine serum (Gibco, 2176398) and 1% penicillin-streptomycin in a 5% CO_2_-humidified incubator at 37 °C. The coding region of SERBP1 was cloned into the pEGFP-N1 vector in-frame with a GFP-tag within the vector. This plasmid was used to generate GFP-tagged SERBP1 mutants, including the following deletants: SERBP1 with deleted amino acids 126 to 137 (Δ126–137, lacking the R domain), Δ163–185 (lacking the RG motif), and Δ361–389 (lacking the RGG-box domain). The Flag-tagged pEnter-SERBP1 plasmid was purchased from Vigene Biosciences (complementary DNA clone MGC: 4404, IMAGE: 2906083) for full-length human SERBP1 (NM_001018067). The GFP-tagged SERBP1 was generated by inserting the coding region into the pEGFP-N1 vector, using the following primers: 5​′-T​CGA​GCT​CAA​GCT​TCG​AAT​TCT​GAT​GCC​TGG​GCACTTACAGG-3′ and 5​′-A​TGG​TGG​CGA​CCG​GTG​GAT​CCC​GAG​CCA​GAG​CTGGGAATGC-3′. The GFP-tagged VCP was generated by inserting the coding region into the pEGFP-N1 vector using the following primers: 5​′-T​CGA​GCT​CAA​GCT​TCG​AAT​TCT​GAT​GGC​CTC​TGGAGCCGATT-3′ and 5​′-A​TGG​TGG​CGA​CCG​GTG​GAT​CCC​GGC​CAT​ACA​GGT​CATCGTCATTG-3′. The pRK5-HA-ubiquitin-WT, pRK5-HA-ubiquitin-K48R, pRK5-HA-ubiquitin-K63R, pRK5-HA-ubiquitin-K48, and pRK5-HA-ubiquitin-K63 plasmids (Addgene 17608, 17604, 17606, 17605, and 17606, respectively) were kindly provided by Prof. Tieshan Tang from Institute of Zoology, Chinese Academy of Science. For stable knockdown experiments, the pLKO.1-puro shRNA vector was obtained from Sigma-Aldrich (SHC001). The pLKO.1-shSERBP1 #1 and #2 were constructed using a specific primer containing a separate targeting sequence (targeting sequence to HSERBP1 1#: 5′-CCTGAAGGTGAAGAACATCAT-3′, HSERBP1 2#: 5′-GCGCTTAAGAAAGAAGGAATA-3′ and MSERBP1 1#: 5′-CCCTTTGAGATTGTAGCATAT-3′, MSERBP1 2#: CCCGTGAAAGAAGATTTGAAA-3′). For transiently knockdown experiments, the siRNA of G3BP1 (5′-UCAACAUGGCGAAUCUUGGUGTT-3′) was transfected by Lipofectamine RNAi Max (Thermo Fisher Scientific, 13778150) according to the manufacturer’s instructions.

### RNA interference and construction of stable cell lines

Lentivirus was produced through transfecting psPAX2 (packaging vector), pMD2.G (envelope vector), and shRNA plasmids into HEK293T cells by PEI MAX (#24765, Polysciences). After 48 h of transfection, the virus-containing medium was harvested. To generate the stable knockdown cells, we infected the HeLa cells using the virus containing either pLKO.1-shHSERBP1 #1, pLKO.1-shHSERBP1 #2, or pLKO.1-control, and 1.5 ml of medium with 1.5 μl of Polybrene (10 μg/ml, Sigma-Aldrich, H9268). After 48 h of transfection, 1 μg/ml puromycin (Sigma-Aldrich, P9620) for HeLa cells or 3 μg/ml for GC-1/GC-2 cells was added to the medium for selection. Stable knockdown cells were identified by Western blotting to confirm the knockdown efficiency after 5 d post-infection.

### Generation of SERBP1 KO mouse model and HeLa cell lines

*Serbp1* KO mice were generated and produced by zygote pronuclear microinjection using CRISPR/Cas9 genome editing as our previously described method [34]. Briefly, 2 single guide RNAs (sgRNAs) (target exon 1 and 3) and Cas9 mRNAs were mixed and injected into C57BL/6 zygotes for generating founder mice. Genomic DNA was extracted from founder mice, followed by Sanger sequencing confirmation and polymerase chain reaction analysis. Founder mice were then backcrossed to WT mice to obtain F1 heterozygous mice and further intercrossed the F1 mice to produce *Serbp1* KO mice for the subsequent experiments.

For *Serbp1* KO cell line generation, CRISPR/Cas9-mediated editing of the SERBP1 genomic locus was performed using either of 2 specific sgRNAs (sgRNA1: 5′- CACCGGAGGCGAATTTTCAGTTGAT-3′; sgRNA2: 5′-CACCGCAGGAAGGCTTCGGCTGCG-3′) and cloned into the lenti-CRISPR vector. The lenti-CRISPR-sgRNA plasmids and empty vector were cotransfected with package plasmids to produce lentivirus, which was subsequently added to the HeLa cell culture medium to infect cells. Single cell clones were picked after selection with puromycin for 2 weeks and expanded for designed experiments.

### Immunofluorescence

Cells or testis cryosections were fixed with 4 % paraformaldehyde in PBS for 1 h at room temperature (RT). Followed by permeabilization with 0.025% Triton X-100, samples were incubated in 5 % bovine serum albumin (BSA) for 1 h at RT and then incubated with primary antibody diluted in BSA overnight at 4 °C. The next day, samples were washed with PBS 3 times (10 min per wash), followed by incubation with Alexa Fluor 488/594 secondary antibodies at RT for 1 to 2 h, and 4,6-diamidino-2-phenylindole was used to visualize the nuclei. Immunofluorescent (IF) images were obtained using a confocal microscope (LSM 900, Zeiss, Germany). The primary antibodies used for IF are summarized as follows: SERBP1 (Abnova, D5C5Z), G3BP1 (Proteintech, 66486-1-AP), TIAR (Proteintech, 66907-1-Ig), PABPC1 (Abcam, Ab21060), 20S (Abcam, ab22674), VCP (Proteintech, 60316-1-lg), FAF2 (Proteintech, 16251-1-AP), ubiquitin (Abcam, Ab7780), K63 ubiquitin (Abcam, Ab179434), and K48 ubiquitin (Abcam, Ab140601).

### SDS-PAGE and Western blotting

Proteins were extracted from cells using lysis buffer (20 mM tris, 1% NP-40, 10% glycerol, 137 mM NaCl, 2 mM EDTA [pH 7.2], 1 mM NaF, 1 mM phenylmethanesulfonyl fluoride, and 1 mM Na_3_VO_4_). Proteins were separated by sodium dodecyl sulfate polyacrylamide gel electrophoresis (SDS-PAGE) gel and then transferred onto polyvinylidene difluoride membranes (IPVH00010, Millipore, USA). The membranes were blocked with 5% fat-free milk for 1.5 h at RT, incubated with the primary antibodies overnight at 4 °C, and subsequently incubated with horseradish peroxidase-conjugated secondary antibodies for 2 h at RT. The proteins were visualized by enhanced chemiluminescence detection (US Everbright Inc, IS0527). The primary antibodies used were listed as follows: SERBP1 (Abnova, D5C5Z), G3BP1 (Proteintech, 66486-1-AP), PSMD10 (Proteintech, 12342-2-AP), PSMA3 (Proteintech, 11887-1-AP), EIF2α (Proteintech, 11170-1-AP), P-EIF2α (Abcam, Ab131505), GFP (Proteintech, 50430-2-AP), hemagglutinin (HA) (Proteintech,51064-2-AP), ubiquitin (Abcam, Ab7780), and α-tubulin (Proteintech, 10727-1-AP). Glyceraldehyde-3-phosphate dehydrogenase (Proteintech, 60004-1) was used as a loading control for normalization.

### Co-IP and mass spectrometry (MS)

For the co-IP of interested proteins, cells were harvested and lysed in 800 μl of lysis buffer (137 mM NaCl, 20 mM tris, 1% NP-40, and 2 mM EDTA [pH = 7.2]) containing protease inhibitors (1x Roche complete protease inhibitor mix) for 30 min on ice. Cell debris was removed by centrifugation at 12,000 g for 20 min, and cell lysates were incubated with either GFP antibody-coupled agarose beads for exogenously expressed GFP-tagged proteins or specific antibodies against detected endogenous proteins at 4 °C overnight, respectively.

For endogenous co-IP, protein A or protein G magnetic beads were added to lysis on the second day and incubated for another 3 h. The beads were washed 3 times with wash buffer, followed by immunoblotting analysis with the indicated antibodies. For the mass spectrometry (MS), immunoprecipitated proteins were separated by SDS-PAGE and then subjected to silver staining to check the efficiency and specificity of the co-IP experiment. Immunoprecipitated proteins were identified using the liquid chromatography-tandem MS approach.

### Silver staining

Proteins on polyacrylamide gels after electrophoretic separation were detected by silver staining according to the manufacturer’s instructions. Briefly, gels were soaked in a fixed solution (10% acetic acid, 50% ethanol, and 40% water) overnight at RT. After rinsing in water, gels were treated with the sensitivity-enhancing solution and stained with silver nitrate solution for 10 min. The gels were then fixed in the development solution for 3 to 10 min until the prospective protein band appeared and stopped development in the stop solution.

### Proteasome activity assay

The proteasome activity was measured as previously described [35]. Briefly, cells were subjected to sodium arsenite (30 min) supplemented with or without the proteasome inhibitor BTZ (Selleck, Houston, TX, USA), rinsed with cold PBS, and lysed on ice for 2 h with lysis buffer (1 mM MgCl_2_, 150 mM NaCl, 1 mM DTT, 1× PhosSTOP tablet, 20 mM Hepes, and 0.5 mM EDTA). Then, 10 μl of cell lysate was resuspended in a 400-μl assay buffer containing 50 μM adenosine triphosphate, 1 mM DTT, and 100 μM Suc-LLVY-AMC (R&D Systems, Wiesbaden, Germany) and transferred to a white opaque polystyrene 96-well plate for 120 min at 37 °C, with excitation wavelength 360 nm and emission wavelength 460 nm on a microplate reader (Synergy HTX, BioTek, USA) for recording.

### TUNEL staining

Apoptotic cells were detected by the One Step TUNEL Apoptosis Assay Kit (Beyotime) following the manufacturer’s instructions. Briefly, the paraffin sections were immersed in tris-HCl (0.1 M, pH = 7.5) containing 3% BSA and 20% fetal bovine serum, followed by dewaxing and hydration. Subsequently, sections were incubated with the TUNEL reaction mixture for 1 h. After staining the nuclei with 4,6-diamidino-2-phenylindole, the signals were captured by a confocal microscope (LSM 900, Zeiss, Germany).

### Histological analysis and imaging

Mouse testes and epididymides were harvested and fixed in Bouin’s solution (Sigma-Aldrich, HT10132) at 4 °C overnight. After dehydration using graded ethanol, samples were embedded in paraffin. Periodic acid-Schiff staining was performed on 5-μm sections using a standard protocol. Images were taken with a light microscope (Axio Scope.A1, Zeiss, Germany).

### Seminiferous tubule microinjection

Eight- to 10-week-old male mice were anesthetized, and the testis was exposed under the standard animal surgery procedure. For microinjection, lentiviral-mediated expression of shRNA targeting *Serbp1* was injected into the seminiferous tubule of the testes using glass needles via the efferent ducts.

### Construction of SG model in vivo

For the heat shock mouse model generation, male mice were anesthetized and immersed in a water bath at 43 °C for 2 h. For the oxidative-stress mouse model creation, male mice were administered sodium arsenite solution (25 mM) in PBS by intratesticular injection. Vehicle control mice were injected with PBS. After waiting for 3 h, the testes were collected, and cryosections were cut to analyze SG formation in vivo using IF assays.

### Colocalization quantification analysis

Pearson’s coefficient was measured to analyze the distributions of SERBP1 and G3BP1 using the Fiji plugin “Coloc2”. *N* = 36 SGs from 12 cells and *N* = 45 SGs from 15 cells. The analysis was performed by selecting three 75 pixels-wide squares per cell.

### Statistical analysis

All experiments were conducted independently 3 times with consistent results. Data were presented as means ± SD, and statistical significances were determined by an unpaired Student *t* test. All statistical analyses were performed using GraphPad Prism8 software. A *P* value less than 0.05 was considered statistically significant. *P < 0.05 and **P < 0.01, ****P < 0.0001.

## Data Availability

All data generated or analyzed during this study are included in this article and/or the Supplementary information files.
