# Supplementary material for: SERBP1 Promotes Stress Granule Clearance by Regulating 26S Proteasome Activity and G3BP1 Ubiquitination and Protects Male Germ Cells from Thermostimuli Damage
Source: Research (Wash D C). 2023 Apr 12;6:0091. doi: 10.34133/research.0091 (PMC10202183; doi:10.34133/research.0091)
Supplement: Supplementary 1 — Figs. S1 to S7. [file research.0091.f1.pdf]

# Supplementary Materials for

## SERBP1 promotes stress granule clearance through regulating 26S proteasome activity and G3BP1 ubiquitination and protects male germ cells from thermo-stimuli damage

Fengli Wang, Lingjuan Wang, Shiming Gan, Shenglei Feng, Sijin Ouyang, Xiaoli Wang, Shuiqiao Yuan

**Figure S1**

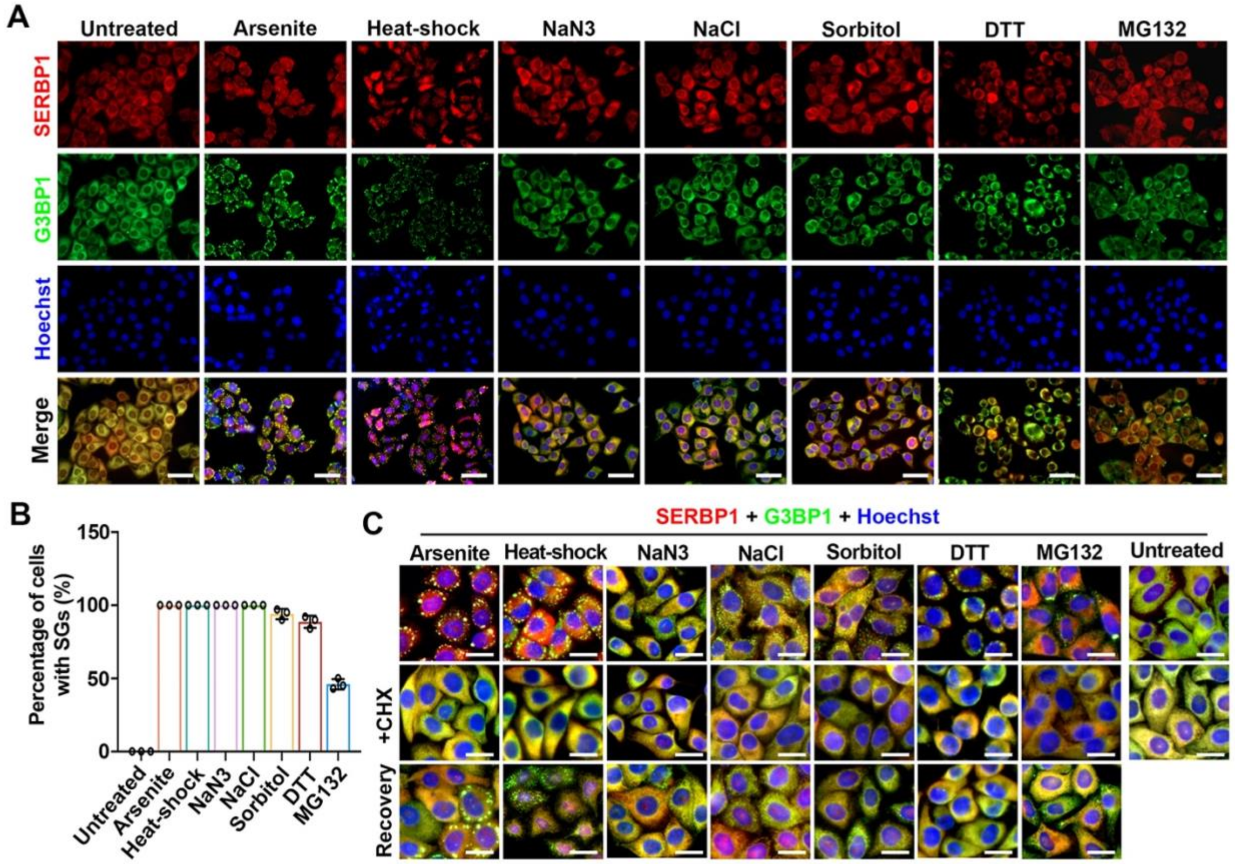


**Figure S1. SERBP1 colocalizes with G3BP1 under various stimuli. (A)** Larger field of confocal images taken with 20× objective of HeLa cells treated with different SGs inducers and stained with anti-G3BP1 (Green) and anti-SERBP1 (Red) antibodies, and counterstained with Hoechst (Blue) are shown. Scale bars = 20 μm. **(B)** Quantification of the percentage of HeLa cells with stress granule induced by various stimuli. Data are collected from three independent experiments and shown as Mean ± SD. **(C)** Confocal images of HeLa cells stained with anti-SERBP1 (Red) and anti-G3BP1 (Green) antibodies and counterstained with Hoechst (Blue) are shown. Cells were either treated with stressors (upper panel, treatment), or co-treated with both stressors and 50 mg/mL cycloheximide (middle panel; CHX), or recovered from SGs induction (lower panel, recovery). Scale bars = 10 μm.

**Figure S2**

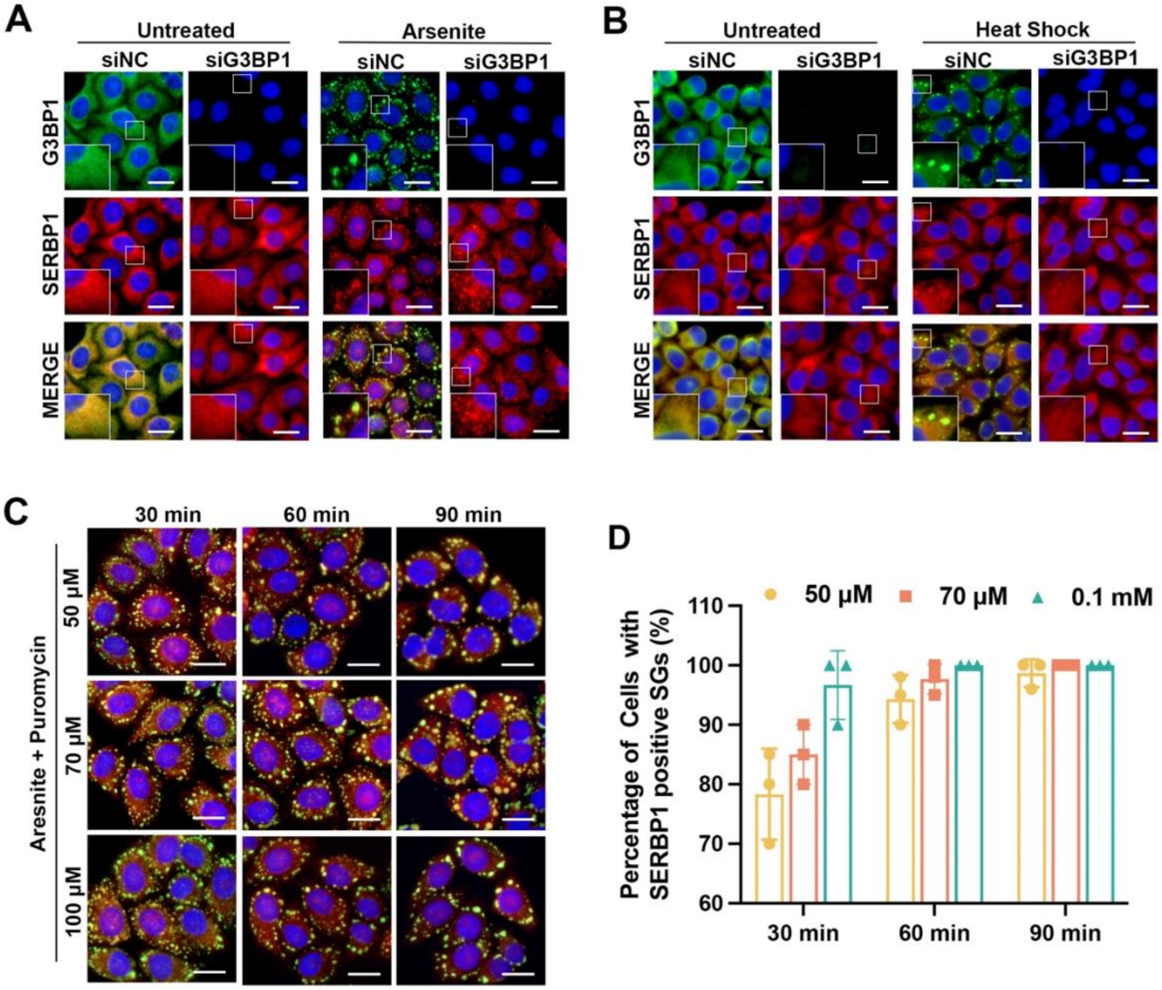


**Figure S2. The association of G3BP1 and SERBP1 during SG assembly. (A-B)** HeLa cells were transfected with the indicated siRNA targeting G3BP1 (siG3BP1) or non-targeted control (siNC) for 48 h, then either subjected to sodium arsenite treatment (0.5 mM, 1 h) (**A**) or incubated at 43℃ for 1 h **(B)**. The SERBP1 (Red) and G3BP1 (Green) were detected by immunofluorescence and visualized by confocal microscopy. Scale bars = 10 μm. **(C)** Confocal images of HeLa cells stained with anti-G3BP1 (Green) and anti-SERBP1 (Red) antibodies and counterstained with Hoechst (Blue). Cells were co-treated with both increased concentrations of arsenite (50 μM, 70 μM, 100 μM,) and puromycin 5 μg/ml, respectively, for different incubation times (30 min, 60 min, 90 min). Scale bars = 10 μm. **(D)** Quantification of the percentage of HeLa cells with SERBP1-positive in conditions tested in (**C**), Data were shown as Mean ± SD from three independent experiments.

**Figure S3**

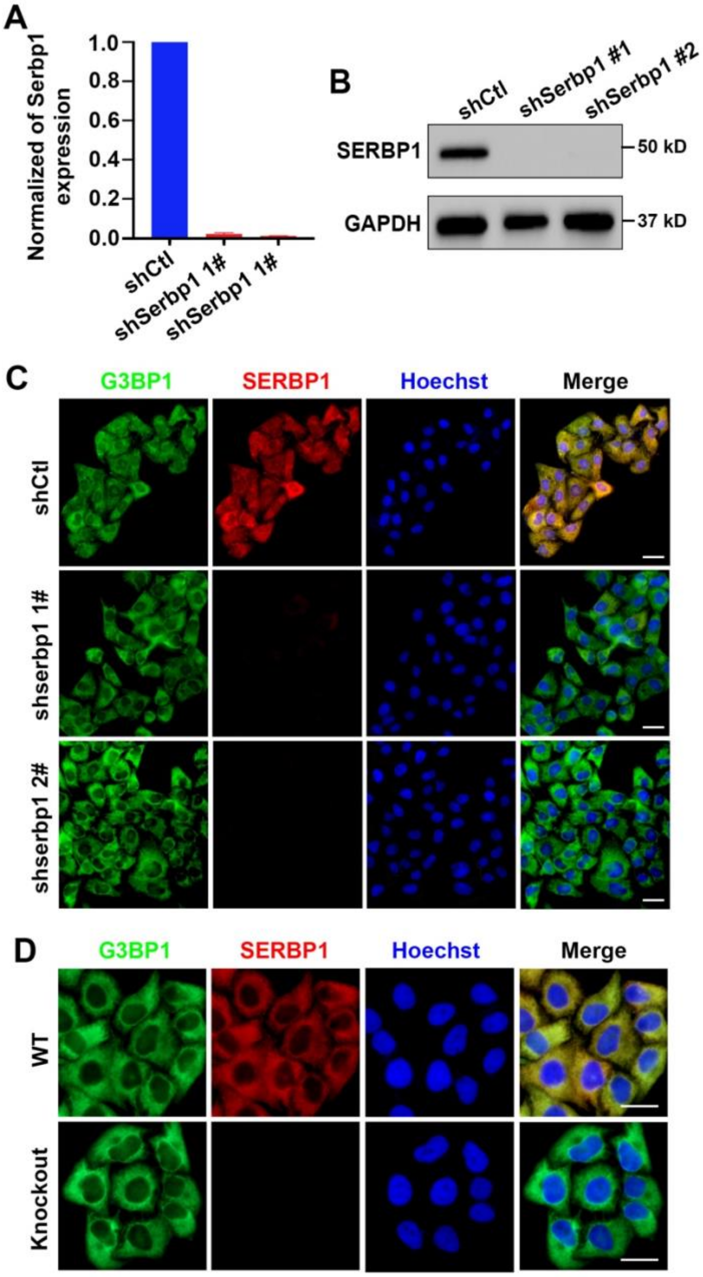


**Figure S3. The verification of SERBP1 knockdown or knockout efficiency in HeLa cells. (A)** RT-qPCR assay analysis of the mRNA levels of *Serbp1* in HeLa cells stably depleted SERBP1 (shSerbp1) using shRNA lentivirus or HeLa cells with control shRNA (shCtl). *Gapdh* was chosen as a normalized control. **(B)** Western blot analysis of SERBP1 protein levels in lysates from HeLa cells stably depleted SERBP1 (shSerbp1) using shRNA lentivirus or HeLa cells with control shRNA (shCtl), GAPDH serves as a loading control. **(C)** Immunofluorescence experiment to detect SERBP1 and G3BP1 protein levels in HeLa cells stably depleted SERBP1 (shSERBP1) using shRNA lentivirus or HeLa cells with control shRNA (shCtl). **(D)** Immunofluorescence experiment to detect SERBP1 and G3BP1 protein levels in WT HeLa (WT) cells or SERBP1 knockout HeLa cells (KO) generated by using CRISPR/Cas9 strategy.

## Figure S4

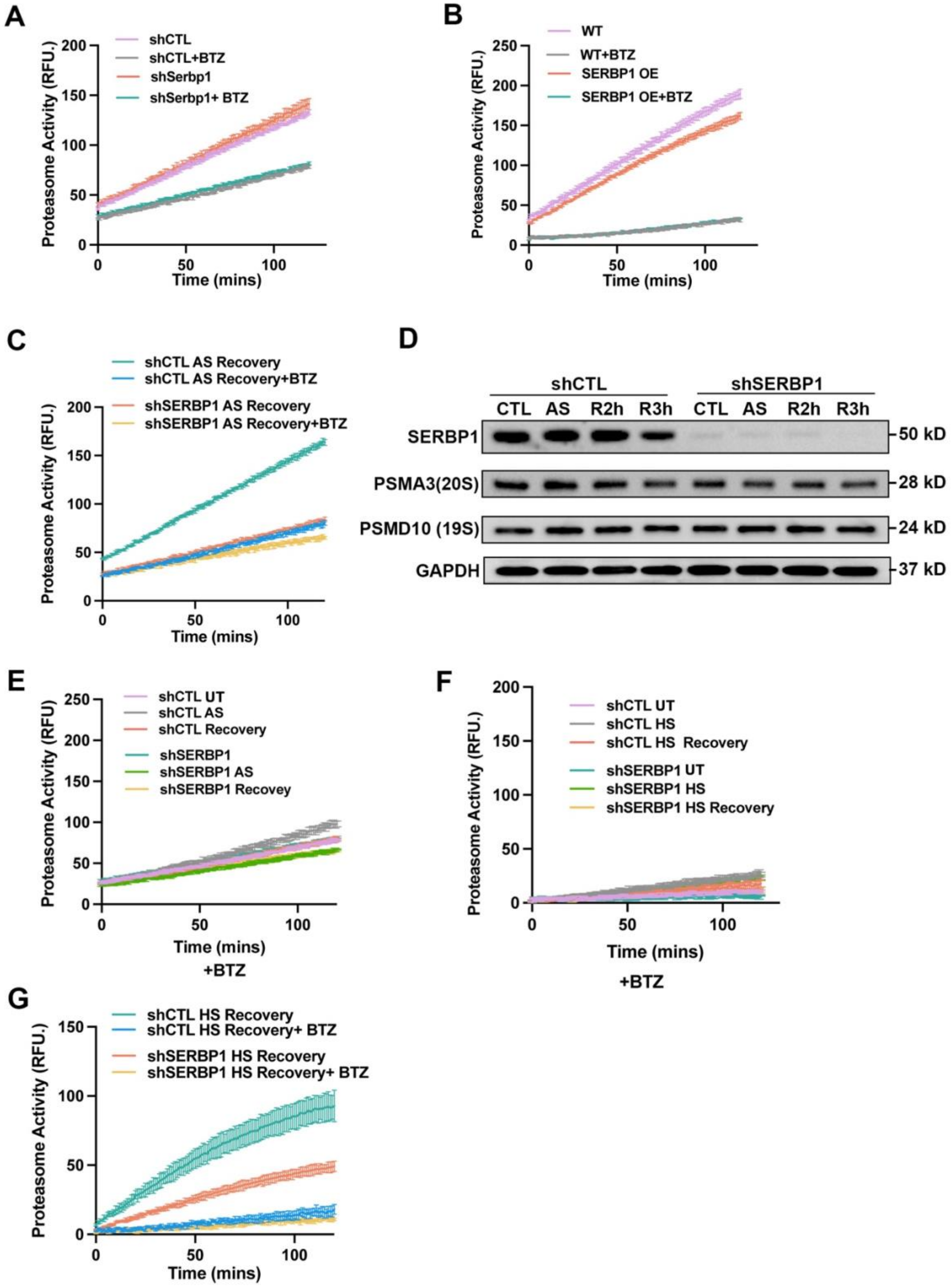
A
shCTL
shCTL+BTZ
shSerbp1
shSerbp1+ BTZ
Proteasome Activity (RFU.)
Time (mins)
B
WT
WT+BTZ
SERBP1 OE
SERBP1 OE+BTZ
Proteasome Activity (RFU.)
Time (mins)
C
shCTL AS Recovery
shCTL AS Recovery+BTZ
shSERBP1 AS Recovery
shSERBP1 AS Recovery+BTZ
Proteasome Activity (RFU.)
Time (mins)
D
shCTL
shSERBP1
CTL AS R2h R3h CTL AS R2h R3h
SERBP1
PSMA3(20S)
PSMD10 (19S)
GAPDH
50 kD
28 kD
24 kD
37 kD
E
shCTL UT
shCTL AS
shCTL Recovery
shSERBP1
shSERBP1 AS
shSERBP1 Recovey
Proteasome Activity (RFU)
Time (mins)
+BTZ
F
shCTL UT
shCTL HS
shCTL HS Recovery
shSERBP1 UT
shSERBP1 HS
shSERBP1 HS Recovery
Proteasome Activity (RFU.)
Time (mins)
+BTZ
G
shCTL HS Recovery
shCTL HS Recovery+ BTZ
shSERBP1 HS Recovery
shSERBP1 HS Recovery+ BTZ
Proteasome Activity (RFU.)
Time (mins)

**Figure S4. SERBP1 modulates 26 proteasome hydrolysis activity. (A)** 26S proteasomal activity measurement using Suc-LLVY-AMC substrate in HeLa cells stably depleted SERBP1 (shSerbp1) using shRNA lentivirus or HeLa cells with control shRNA (shCtl), while each group was co-treated with Bortezomib (BTZ) as corresponding negative control. Cells were then lysed and AMC signals were detected with excitation wavelength 360 nm and emission wavelength 460 nm on a microplate reader to record for 120 min. **(B)** 26S proteasomal activity measurement using Suc-LLVY-AMC substrate in HeLa cells overexpressing SERBP1 (SERBP1 OE) or WT cells transfected with empty vector (WT), while each group was co-treated with Bortezomib as corresponding negative control. Cells were then lysed and AMC signals were detected with excitation wavelength 360 nm and emission wavelength 460 nm on a microplate reader to record for 120 min. **(C)** 26S proteasomal activity measurement using Suc-LLVY-AMC substrate. HeLa cells stably depleted SERBP1 (shSERBP1) using shRNA lentivirus or HeLa cells with control shRNA (shCtl) were treated with 0.5 mM arsenite for 60 min (AS) then allowed to recover for 120 min after drugs removal (Recovery), while each group was co-treated with Bortezomib (BTZ) as the corresponding negative control. Cells were lysed and AMC signals were detected with excitation wavelength 360 nm and emission wavelength 460 nm on a microplate reader to record for 120 min. **(D)** Protein levels of PSMD10 and PSMA3 in indicated HeLa cells. HeLa cells stably depleted SERBP1 (shSERBP1) using shRNA lentivirus or HeLa cells with control shRNA (shCTL) were treated with 0.5 mM arsenite for 60 min (AS) then allowed to recover for 2 h (R2h) or 3 h (R3h) after drugs removal. Cell lysis was prepared and immunoblotted for SERBP1, PSMD10, and PSMA3, while GAPDH serves as a loading control. **(E)** 26S proteasomal activity measurement using Suc-LLVY-AMC substrate. HeLa cells stably depleted SERBP1 (shSERBP1) using shRNA lentivirus or HeLa cells with control shRNA (shCTL) were cultured at 43℃ for 1 h, and allowed to recover for 30 min (HS Recovery), while each group was co-treated with Bortezomib as corresponding negative control. Cells were lysed and AMC signals were detected with excitation wavelength 360 nm and emission wavelength 460 nm on a microplate reader to record for 120 min. **(F)** 26S proteasomal activity measurement using Suc-LLVY-AMC substrate. HeLa cells stably depleted SERBP1 (shSERBP1) using shRNA lentivirus or HeLa cells with control shRNA (shCTL) were treated with 0.5 mM arsenite for 60 min (AS), and allowed to recover for 120 min after drugs removal (Recovery), while all these groups were co-treated with Bortezomib (BTZ), then lysed and AMC signals were detected with excitation wavelength 360 nm and emission wavelength 460 nm on a microplate reader to record for 120 min. Related to **Figure 4F. (G)** 26S proteasomal activity measurement using Suc-LLVY-AMC substrate. HeLa cells stably depleted SERBP1 (shSERBP1) using shRNA lentivirus or HeLa cells with control shRNA (shCTL) were cultured at 43°C for 1 hr (heat shock, HS), and allowed to recover for 30 min (HS Recovery), while all these groups were co-treated with Bortezomib (BTZ), then lysed and AMC signals were detected with excitation wavelength 360 nm and emission wavelength 460 nm on a microplate reader to record for 120 min. Related to **Figure 4H.**

**Figure S5**

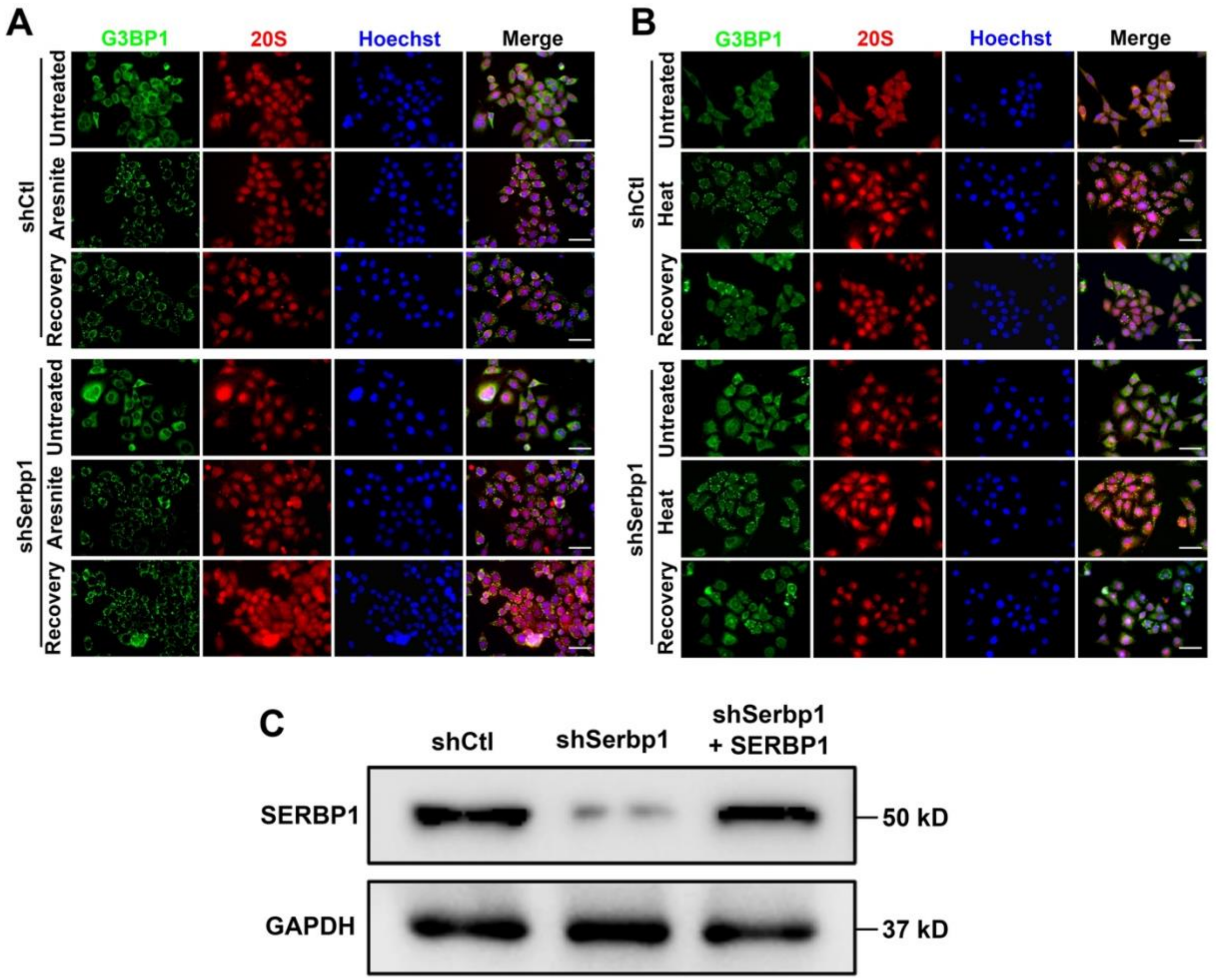


**Figure S5. SERBP1 regulates 20S proteasomal localization in SGs. (A-B)** Larger field of confocal images taken with 20× objective. HeLa cells stably depleted SERBP1 (shSERBP1) using shRNA lentivirus or HeLa cells with control shRNA (shCtl) were treated with 0.5 mM sodium arsenite for 1 h (Arsenite) and allowed to recover for 60 min (Recovery) (**A**), or cultured at 43°C for 1 h (Heat shock) and allowed to recover for 60 min (Recovery) (**B**), then stained with anti-G3BP1 and anti-20S antibodies. Nuclei were counterstained with Hoechst. Scale bars = 20 μm. **(C)** Protein level of SERBP1 in indicated HeLa cell lysates. Lysis from HeLa cells stably depleted SERBP1 (shSERBP1) using shRNA lentivirus or SERBP1 depleted HeLa cells transfected with plasmid encoding SERBP1 (shSERBP1+SERBP1), or HeLa cells with control shRNA (shCtl) was prepared and immunoblotted for SERBP1, while GAPDH serves as a loading control.

**Figure S6**

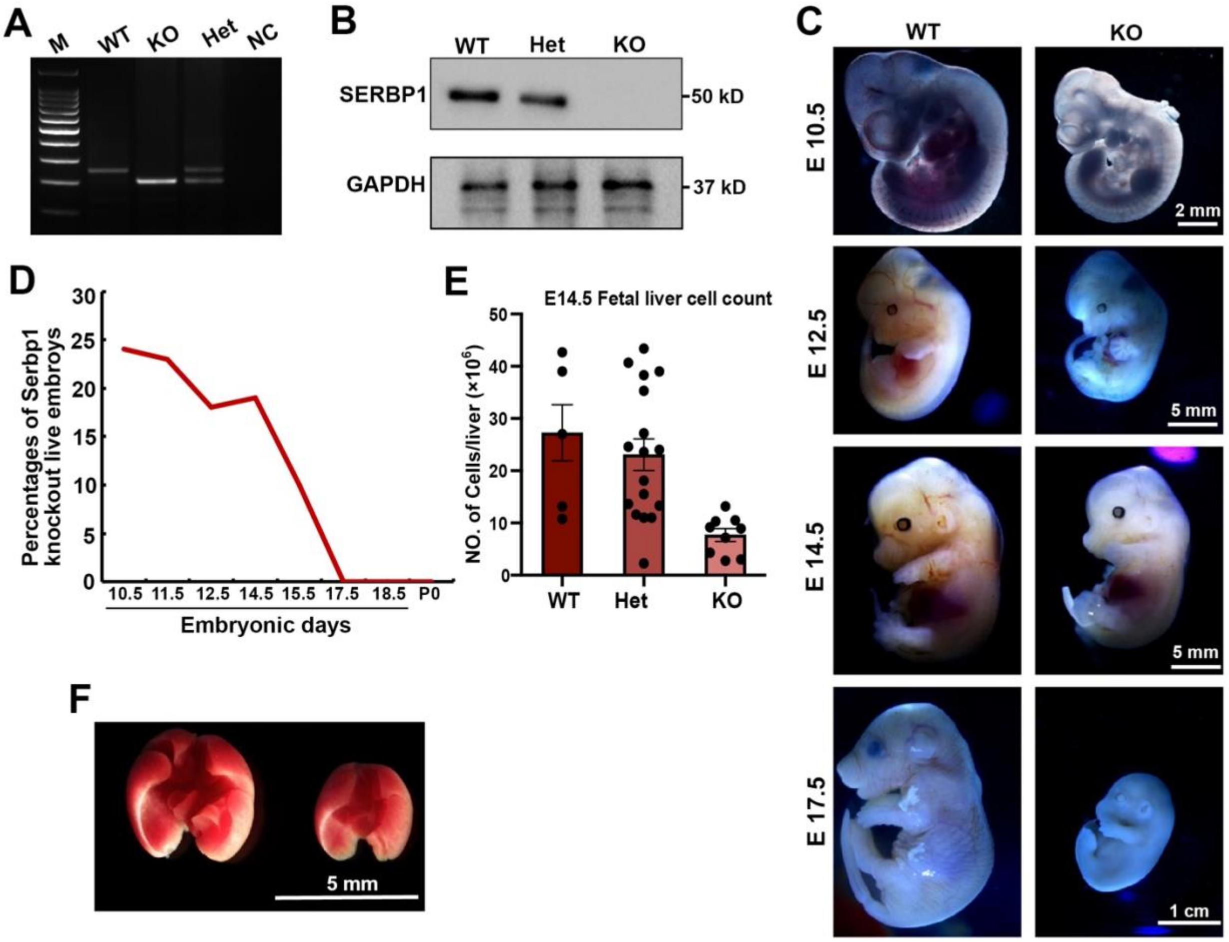


**Figure S6. Serbp1-deficiency in mice leads to embryonic lethality. (A)** DNA isolated from mouse embryos (E14.5) was genotyped by PCR. NC, negative control. **(B)** Western blot analysis of Serbp1 protein levels in lysates from mouse embryos with indicated genotypes. GAPDH serves as a loading control. **(C)** Gross morphology of WT and *Serbp1* KO embryos at E10.5, E12.5, E14.5 and E17.5. Scale bars are shown as indicated. **(D)** Gradual loss of *Serbp1* KO embryos during mouse gestation. *Serbp1* heterozygous mice were intercrossed. Genotypes of offspring were determined at different gestational days or after birth, and the percentage of *Serbp1* KO live embryos was shown as indicated. **(E)** Total number of fetal liver cells from *Serbp1* WT, *Serbp1* heterozygous and KO embryos at E14.5. **(F)** Gross morphology of representative fetal livers from *Serbp1* WT and KO embryos at E14.5. Scale bars = 5 mm.

## Figure S7

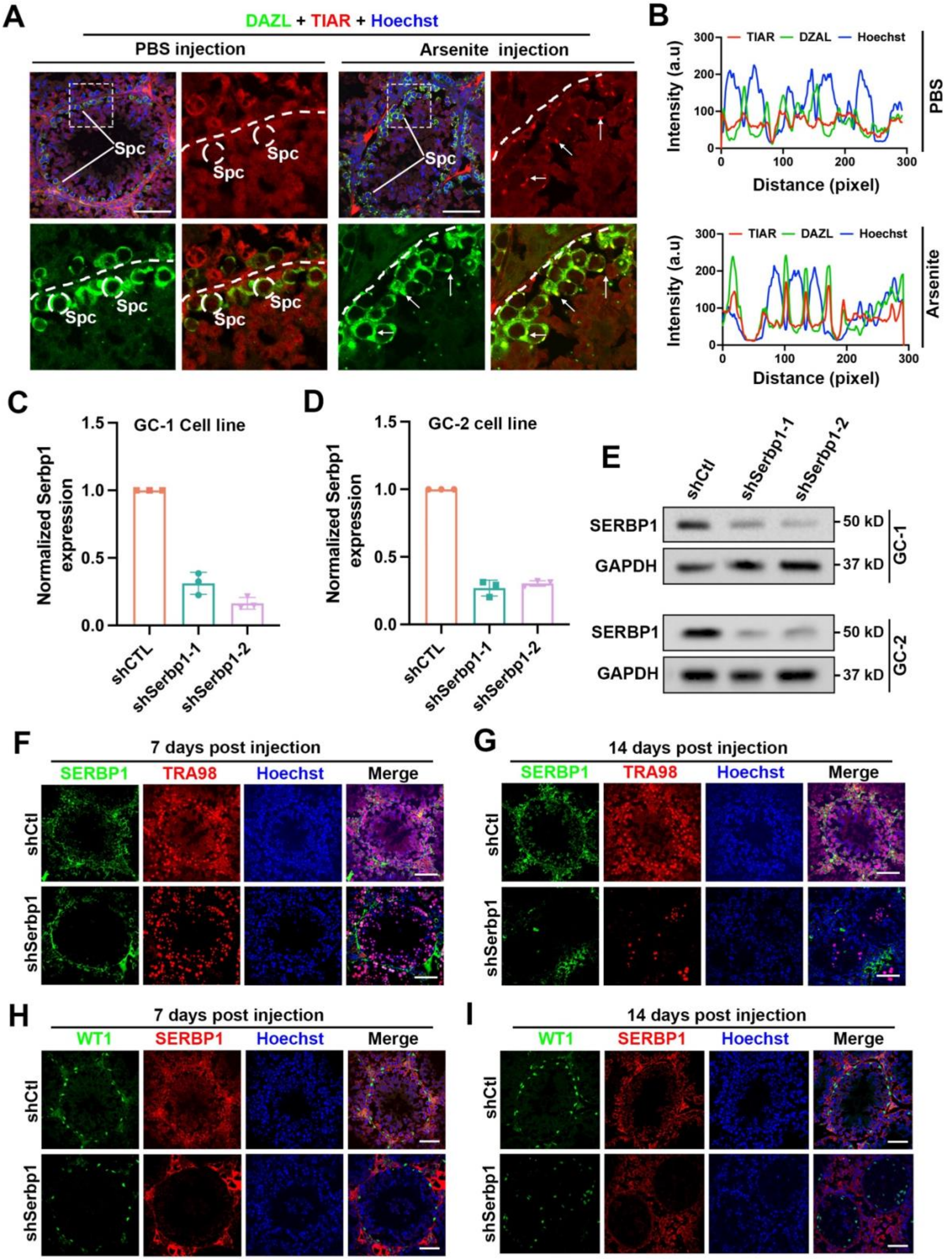

A
DAZL + TIAR + Hoechst
PBS injection
Arsenite injection
Spc
B
TIAR DZAL Hoechst
Intensity (a.u)
Distance (pixel)
PBS
TIAR DAZL Hoechst
Arsenite
C
GC-1 Cell line
Normalized Serbp1 expression
shCTL
shSerbp1-1
shSerbp1-2
D
GC-2 cell line
E
shCtl
shSerbp1-1
shSerbp1-2
SERBP1
GAPDH
50 kD
37 kD
GC-1
GC-2
F
7 days post injection
SERBP1
TRA98
Hoechst
Merge
shCtl
shSerbp1
G
14 days post injection
H
WT1
I

**Figure S7. SERBP1 deficiency in testis impairs germ cell development under heat shock stimuli. (A)** Representative immunofluorescence (IF) images of testis sections stained for DAZL and the general SG marker TIAR are shown. Localization of SERBP1 and G3BP1 in mouse testes injected either with PBS or sodium arsenite. The dotted cycles indicate spermatocytes (Spc). Scale bars = 50 μm. **(B)** Signal profiles to analyze co-localization of DZAL and TIAR between PBS injection and arsenite injection groups using Image J software. The graph corresponds to the normalized pixel-by-pixel gray value. **(C-D)** RT-qRT to determine SERBP1 mRNA levels in GC-1 (**C**) and GC-2 (**D**) cell lines transacted with control shRNA (shCtl) or SERBP1 shRNAs (shSerbp1-1 and shSerbp1-2). GAPDH serves as an internal control. **(E)** Western blot analysis of SERBP1 protein levels in GC-1 (upper panels) and GC-2 (lower panels) cells transacted with control shRNA or SERBP1 shRNAs. GAPDH serves as a loading control. **(F-G)** Representative images showing co-immunostaining for germ cell marker TRA98 (Red) and SERBP1 (Green) of control and SERBP1 knockdown mouse testes. 7 days **(F)** or 14 days post injection **(G)**, testes were collected and cryosections were made accordingly. Scale bars = 50 μm. **(H-I)** Representative images showing co-immunostaining for Sertoli cell marker WT1 (Green) and SERBP1 (Red) of control and SERBP1 knockdown mouse testes. 7 days **(H)** or 14 days post injection **(I)**, testes were collected and cryosections were made accordingly. Scale bars = 50 μm.
